# Enhancing singlet oxygen generation in semiconducting polymer nanoparticles through fluorescence resonance energy transfer for tumor treatment†
†Electronic supplementary information (ESI) available: Details of general experimental information, NMR and MS spectra. See DOI: 10.1039/c8sc05501g


**DOI:** 10.1039/c8sc05501g

**Published:** 2019-04-11

**Authors:** Jiayang Jiang, Yuanyuan Qian, Zihan Xu, Zhuang Lv, Peng Tao, Mingjuan Xie, Shujuan Liu, Wei Huang, Qiang Zhao

**Affiliations:** a Key Laboratory for Organic Electronics and Information Displays , Institute of Advanced Materials (IAM) , Jiangsu National Synergetic Innovation Center for Advanced Materials (SICAM) , Nanjing University of Posts and Telecommunications (NUPT) , Nanjing 210023 , P. R. China . Email: iamsjliu@njupt.edu.cn ; Email: iamqzhao@njupt.edu.cn; b Shaanxi Institute of Flexible Electronics (SIFE) , Northwestern Polytechnical University (NPU) , Xi'an 710072 , Shaanxi , China . Email: provost@nwpu.edu.cn

## Abstract

Photosensitizers (PSs) are of particular importance for efficient photodynamic therapy (PDT). Challenges for PSs simultaneously possessing strong light-absorbing ability, high ^1^O_2_ generation by effective intersystem crossing from the singlet to the triplet state, good water-solubility and excellent photostability still exist. Reported here are a new kind of dual-emissive semiconducting polymer nanoparticles (SPNs) containing fluorescent BODIPY derivatives and near-infrared (NIR) phosphorescent iridium(iii) complexes. In the SPNs, the BODIPY units serve as the energy donors in the fluorescence resonance energy transfer (FRET) process for enhancing the light absorption of the SPNs. The NIR emissive iridium(iii) complexes are chosen as the energy acceptors and efficient photosensitizers. The ionized semiconducting polymers can easily self-assemble to form hydrophilic nanoparticles and homogeneously disperse in aqueous solution. Meanwhile, the conjugated backbone of SPNs provides effective shielding for the two luminophores from photobleaching. Thus, an excellent overall performance of the SPN-based PSs has been realized and the high ^1^O_2_ yield (0.97) resulting from the synergistic effect of BODIPY units and iridium(iii) complexes through the FRET process is among the best reported for PSs. In addition, owing to the phosphorescence quenching of iridium(iii) complexes caused by ^3^O_2_, the SPNs can also be utilized for O_2_ mapping *in vitro* and *in vivo*, which assists in the evaluation of the PDT process and provides important instructions in early-stage cancer diagnosis.

## Abstract 2

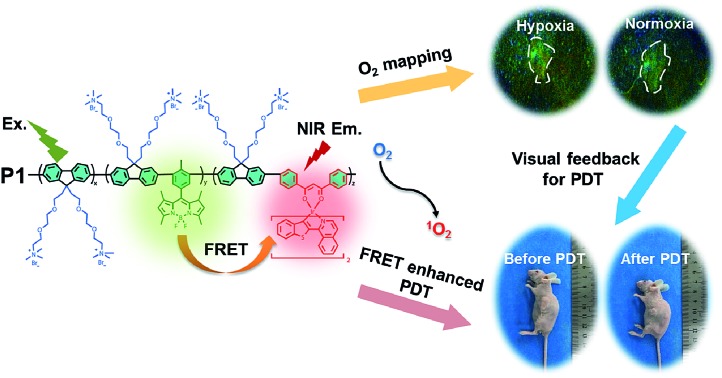
We have developed semiconducting polymer nanoparticle-based photosensitizers for O_2_ mapping and enhanced the PDT effect by using fluorescence resonance energy transfer.

## Introduction

Photodynamic therapy (PDT) with high optical precision has attracted tremendous attention as an emerging clinical modality for treating cancers, which brings little damage to normal tissues, no wound surface, and negligible long-term sequelae or immune attack.^1^,^2^ During the PDT process, photosensitizers (PSs), which convert ^3^O_2_ to ^1^O_2_ or other reactive oxygen species (ROS) under light irradiation, play a crucial role in the irreversible cytotoxic process to cancer cells.^3^,^4^ In order to achieve effective PDT, high ^1^O_2_ production *via* an effective intersystem crossing (ISC) from the singlet (S_1_) to the triplet state (T_1_), intense light absorption, good photostability and water-solubility should be required for PSs. Currently, PSs are mainly based on organic dyes and phosphorescent transition-metal complexes (PTMCs), however, few of these PSs can simultaneously meet the above requirements. For PSs based on organic dyes, such as BODIPY, fluorescein and methylene blue, they have strong light absorption, but usually cannot achieve highly efficient ^1^O_2_ generation because of their weak ISC from S_1_ to T_1_.^5^–^7^ In addition, their photostability is usually poor for long-time PDT treatment. PTMCs are among the most promising candidates for PSs, owing to their high ^1^O_2_ quantum yield and excellent photostability.^8^ However, suffering from the spin-forbidden triplet transition, light absorption in visible regions of PTMCs is usually not strong enough for highly efficient PDT. Moreover, the rigid structures of these PSs are usually hydrophobic, which will induce aggregation in physiological environments and lead to a remarkable reduction of their luminescence intensity and ^1^O_2_ generation. Therefore, it still remains the challenge to develop photosensitizers with excellent overall performance.

To address the above-mentioned issues, herein, we proposed an effective strategy of light-harvesting fluorescence resonance energy transfer (FRET) to design excellent photosensitizers based on semiconducting polymer nanoparticles (SPNs) to amplify the PDT efficiency. To demonstrate the effectiveness of this strategy, BODIPY units and iridium(iii) complexes were simultaneously introduced into the backbone of cationic polyfluorene. Among them, BODIPY units served as the energy donors with a high extinction coefficient in the FRET process for enhancing the light absorption of the SPN-based PSs.^9^,^10^ The NIR emissive iridium(iii) complexes with a suitable triplet energy level were chosen as the energy acceptors and photosensitizers in SPNs, which can easily transfer the energy to the ground state of O_2_ and lead to a high ^1^O_2_ quantum yield. Moreover, the ionized side chains of the polyfluorene backbone endowed the semiconducting polymers with the capability to form hydrophilic nanoparticles through self-assembly and homogeneously disperse in aqueous solution for further applications. Meanwhile, the conjugated backbone of SPNs provided an efficient shielding effect for the two luminophores from photobleaching, improving the photostability.^11^ Attributed to the rational structural design, together with the synergistic effect of BODIPY units and iridium(iii) complexes through a highly efficient FRET process, a high ^1^O_2_ quantum yield (0.97) of SPNs has been achieved, which is among the best reported for PSs. In addition, owing to the phosphorescence quenching of iridium(iii) complexes caused by oxygen through the energy transfer process, the SPNs could also be used for O_2_ mapping *in vitro* and *in vivo*, which assisted in the evaluation of the PDT process and provided important guidance in early-stage cancer diagnosis.^12^

## Results and discussion

### Design, synthesis and characterization of SPNs

**Scheme 1:**
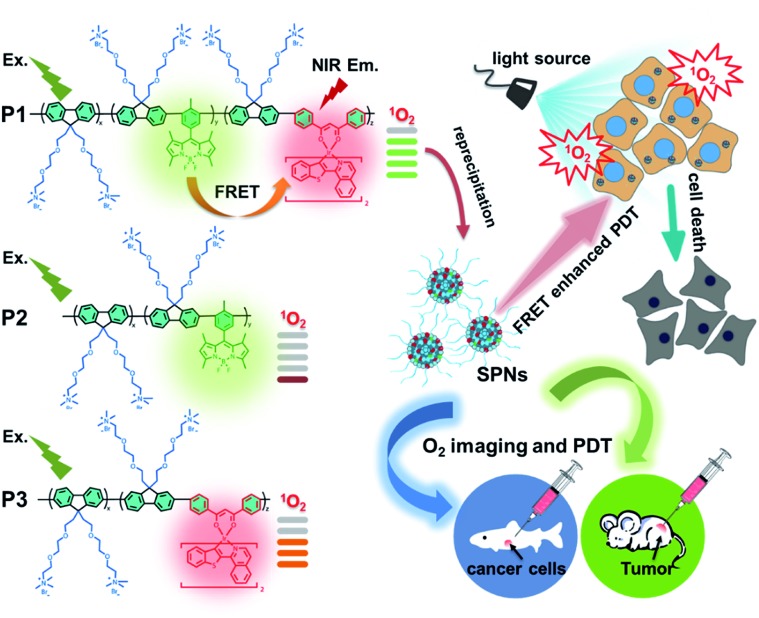
Structures of **P1**, **P2** and **P3** and graphical representation of **P1** used as photosensitizers with enhanced ^1^O_2_ generation through fluorescence resonance energy transfer for efficient tumor treatment.

**Fig. 1:**
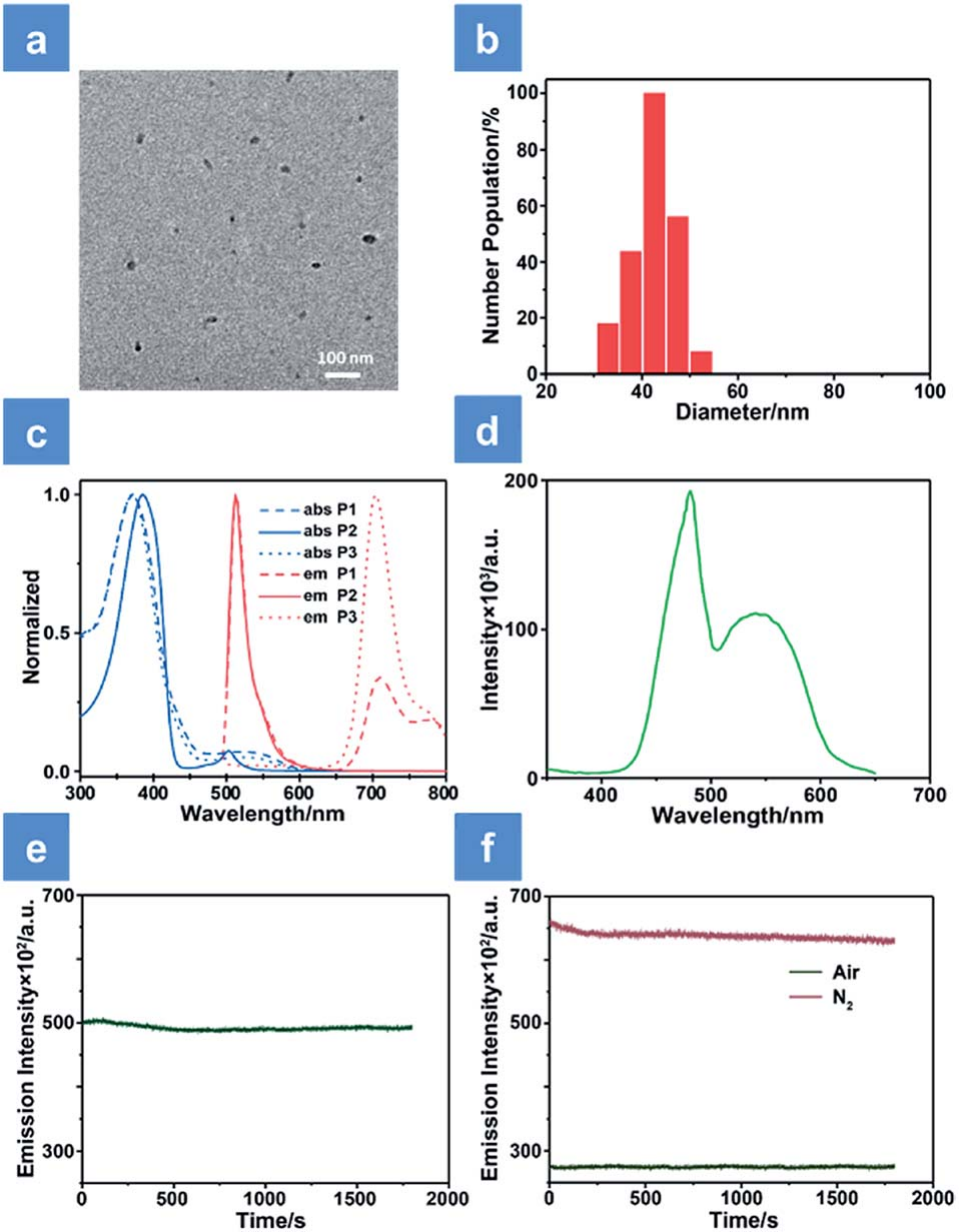
Characterization of SPNs. (a) TEM image of **P1** in aqueous solution. (b) Dynamic light scattering column diagram of **P1**. (c) The absorption and emission spectra of **P1**, **P2** and **P3** in aqueous solution. (d) The excitation spectrum of **P1** monitored at 704 nm. (e) Photostability examination of **P1** monitored at 515 nm under normoxia conditions. (f) Photostability examination of **P1** monitored at 704 nm under normoxia and hypoxia conditions. A xenon lamp with a power of 30 mW cm^–2^ was used in evaluating the photostability.

As shown in Scheme 1, we rationally designed and synthesized polyfluorene-based dual-emissive SPNs containing BODIPY units and NIR emissive iridium(iii) complexes by the Suzuki coupling reaction. The large π skeleton constructed iridium(iii) complex monomers (**M1**) were prepared using a two-step process (see ESI†). Meanwhile, the green-emitting BODIPY monomers (**M2**) were prepared according to the literature report.^13^ The synthesis of semiconducting polymers is illustrated in Scheme S1.† The polymers were precipitated in the mixture of methanol and H_2_O (v/v = 10 : 1), and then treated by Soxhlet extraction for 3 days. After quaternization with trimethylamine, **P1** was obtained. The ionized side chain of **P1** improved the water-solubility, avoiding luminescence quenching caused by aggregation. In addition, **P2** and **P3** were also prepared according to the same procedure for comparison. As seen in Table S1,† the real contents of **M1** and **M2** units in SPNs estimated by ^1^H NMR spectra were 10.1% and 4.8%, respectively, which were lower than those in feeding ratio probably because of the reaction activity and steric hindrance. The weight-average molecular mass (*M*_w_) of **P1**, **P2** and **P3** was 27 300, 11 800 and 15 500 with polydispersity indexes (PDI) of 1.26, 1.67 and 1.10, respectively. The SPNs were constructed under ultrasound treatment in water and a small fraction of large-sized particles were removed *via* a 0.22 μm membrane. As shown in Fig. 1a, transmission electron microscopy (TEM) revealed the uniform morphology of **P1**-based SPNs. Additionally, dynamic light scattering (DLS) showed the hydrodynamic diameter of SPNs to be approximately 40 nm (Fig. 1b) in phosphate buffered solution (PBS). The *ζ*-potential of SPNs in deionized water is 36.8 mV. The suitable size and charged surface suggested that the nanoparticles could be well dispersed in aqueous solution.

### Photophysical properties of SPNs

The ultraviolet-visible absorption and photoluminescence (PL) spectra of all monomers and SPNs have been studied (Fig. 1 and S1†). As shown in Fig. 1c, an intense absorption band between 300 and 440 nm is observed for **P1**, **P2** and **P3**. This band corresponds to the π–π* transitions of conjugated backbones. A sharp absorption band of **P2** appears between 480 and 520 nm, which is assigned to the characteristic S_0_–S_1_ transition of **M2** units. By contrast, the relatively wide and weak absorption between 460 and 580 nm of **P3** is attributed to the metal-to-ligand charge transfer (MLCT) transition of **M1** units. For the absorption of **P1**, the broad band in the range from 490 to 600 nm is attributed to the overlap between the MLCT absorption of **M1** units and characteristic transition of **M2** units. Then, the PL spectrum of SPNs has been investigated upon excitation at 488 nm. From the spectrum we can see that **P2** has a strong emission peak centered at 515 nm, which originates from the emission of **M2** units. **P3** exhibits a broad near-infrared emission in the range of 650–800 nm with a quantum yield of 0.13, which is attributed to the phosphorescence emission of **M1**. As expected, **P1** displays two emission peaks from both **M1** and **M2** units and the quantum yield between 650 and 800 nm is 0.11. The NIR emission of iridium(iii) complexes could enhance the penetration depth, reduce the tissue scattering and be far less likely to damage the healthy tissue.^14^

Moreover, as seen from Fig. S1,† the emission of **M2** (500–570 nm) has a good overlap with the absorption of **M1** (490–600 nm). This result indicates that **M1** and **M2** are capable of forming an efficient FRET pair. To validate the energy transfer between **M1** and **M2**, the excitation spectrum of **P1** monitored at 704 nm is acquired (Fig. 1d). The sharp signal around 480 nm is assigned to the excitation of **M2** units and the broad peak from 500–600 nm belongs to the **M1** units. This spectral result demonstrates that BODIPY units have given assistance to the excitation process and is beneficial to efficient energy transfer. In order to calculate the energy transfer efficiency, the luminescence lifetime measurement (monitored between 500 and 550 nm) of **P1** and **P2** has been conducted. The lifetimes of **M2** units in **P1** and **P2** have been summarized in Table S1.† The efficiency of energy transfer between **M1** and **M2** in **P1** was calculated to be 51% according to the following eqn (1):^15^1
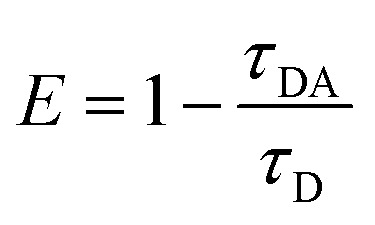
where *τ*_DA_ is the fluorescence lifetime value of the donor when the acceptor exists and *τ*_D_ is the fluorescence lifetime of the donor when the acceptor is absent. The relatively high energy transfer rate facilitates the enlargement of light utilization *via* enhancing the absorption between 490 and 600 nm of **P1**.^9^

In addition, anti-photobleaching ability is an important factor for biomedical applications. We evaluated the photostability of **P1** in aqueous solution under irradiation of a 488 nm laser for 30 min (Fig. 1e and f). As the time increased, the two emission bands of **P1** remained over 90% of the initial intensity. This result demonstrates that the photostability of BODIPY units has been improved owing to the shielding effect of the conjugated backbone.^11^

### ^1^O_2_ generation by SPNs

The difference of ^1^O_2_ generation ability in aqueous solution among **P1**, **P2** and **P3** is discussed as follows. The monitoring of ^1^O_2_ generation was carried out *via* the 9,10-anthracenediylbis(methylene) malonic acid (ABDA) assay.^16^ From Fig. S2† we can see that the order of the absorption quenching rate was **P1** > **P3** > Ru(ii)(bpy)_3_^2+^ > **P2**, suggesting that **P1** is the best photoactivatable ^1^O_2_ producer among the SPNs. Then, Ru(ii)(bpy)_3_^2+^ (*Φ*_Δ_ = 0.41 in water) was utilized as the reference for calculating the ^1^O_2_ yield of SPNs, which was obtained by using the absorbance change (Δ*A*) of ABDA within 9 min according to eqn (2):^17^2
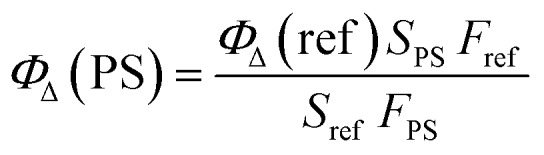
where **P1** and Ru(bpy)_3_^2+^ are expressed as PS and reference, respectively. *S* refers to the slope of the fitting curve of ABDA absorption difference (400 nm) and irradiation time. *F* equals 1 – 10^–OD^ (OD values are the optical density of **P1** or Ru(bpy)_3_^2+^ at 475 nm).

**Scheme 2:**
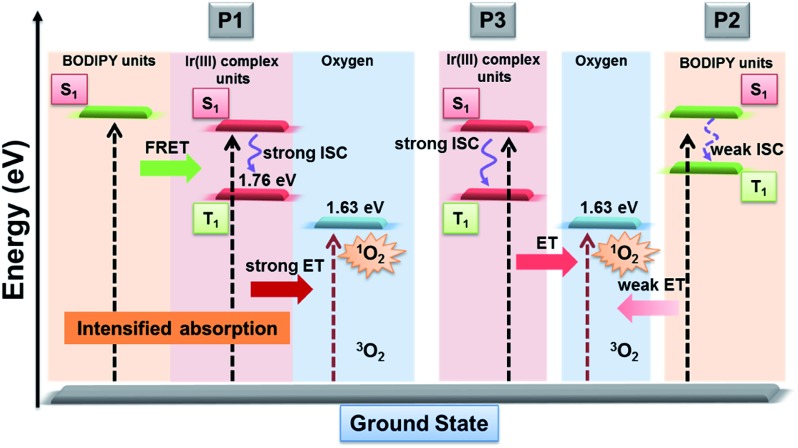
Schematic illustration of SPNs for enhanced ^1^O_2_ generation.

As is known that ^1^O_2_ generation depends on the energy transfer between the energy donor and acceptor, the difference in energy levels between the triplet state of iridium(iii) complex units and the excited state of molecular oxygen largely determines the final ^1^O_2_ yield.^18^ According to the PL spectrum of SPNs, the triplet energy of iridium(iii) complexes (1.76 eV) in **P3** matches well with the energy of the excited state of molecular oxygen (1.63 eV), leading to a high ^1^O_2_ yield (0.78) of **P3**. In contrast, **P2** without iridium(iii) complexes shows a relatively low ^1^O_2_ yield (0.12), because BODIPY derivatives without electron donors or heavy atoms cannot possess efficient intersystem crossing between S_1_ and T_1,_ which results in the low ^1^O_2_ generation.^6^ By contrast, the FRET of **P1** can enhance the light absorption ability as well as giving rise to the highest ^1^O_2_ generation yield (0.97), which will improve the cancer-killing efficiency during PDT (Scheme 2).^9^

### Luminescence response to oxygen in aqueous solution

**Fig. 2:**
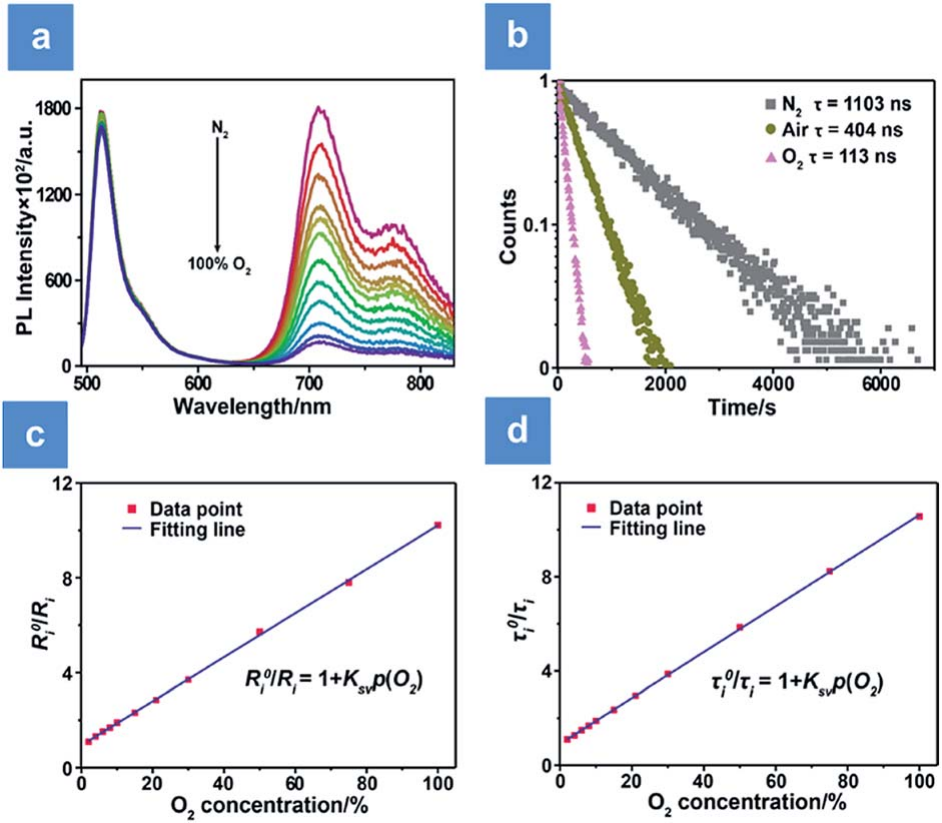
PL intensity and lifetime variations of **P1** under hypoxia conditions. (a) PL spectra of **P1** at different oxygen levels (0, 2, 4, 6, 8, 10, 15, 21, 30, 50, 75 and 100%) and under excitation at 488 nm. (b) Luminescence decay curves of **P1** collected at 704 nm. (c) The functional relationship between *R*0i/*R*_i_ and O_2_ content (*R*^2^ = 0.998, *K*_SV_ = 0.097%^–1^). (d) The functional relationship between *τ*_0_/*τ* and O_2_ content (*R*^2^ = 0.996, *K*_SV_ = 0.093%^–1^). The concentration of the SPNs in solution is 70 μg mL^–1^.

The oxygen sensing experiments of **P1** have been conducted in PBS at the concentration of 70 μg mL^–1^. The relationship between PL intensity and O_2_ contents is shown in Fig. 2a. Under 488 nm excitation, the PL intensity at 515 nm of **P1** remains unchanged at different O_2_ contents. In contrast, the NIR signal of **P1** collected at 704 nm increases dramatically along with the decrease of O_2_ content. As a result, efficient ratiometric luminescence O_2_ sensing of the SPNs has been realized based on the two luminophores. And the large wavelength difference (∼200 nm) between the emission of **M1** and **M2** in **P1** is beneficial for two intensity-based non-interfering O_2_ sensing. To quantitatively evaluate the capability of **P1** for oxygen detection, the Stern–Volmer equation (eqn (3)) is defined as follows:^19^3
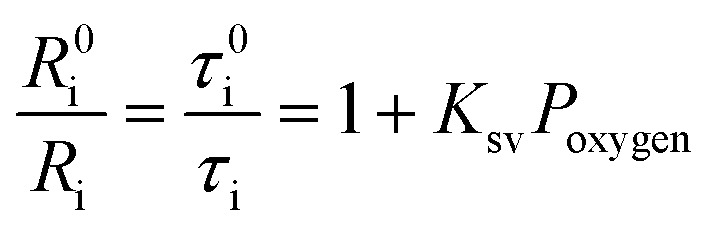
where *R*_i_ and *R*0i are defined as the intensity ratios (*I*_704 nm_/*I*_515 nm_) at different oxygen contents and in nitrogen, respectively. *τ*_i_ and *τ*0i are defined as the phosphorescence lifetimes at 704 nm at various oxygen concentrations and in nitrogen, respectively. *K*_SV_ is the Stern–Volmer constant, and *P*_oxygen_ is the oxygen partial pressure. The intensity ratio (*I*_704 nm_/*I*_515 nm_) shows a linear relationship with different oxygen partial pressures. As illustrated in Fig. 2c, O_2_ triggered an evident spectral change and a notable 10.6 fold enhancement of the intensity ratio was obtained. By using eqn (4) below, the oxygen sensitivity of **P1** can be figured out:^20^4
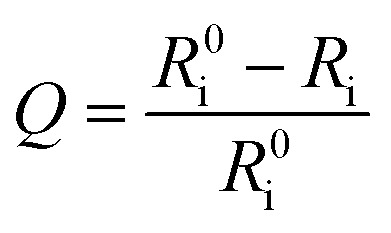
where *Q* is the quenching efficiency value, and *R*_i_ and *R*0i are the emission intensity ratio (*I*_704 nm_/*I*_515 nm_) at different oxygen contents and in nitrogen, respectively. As a result, the quenching efficiency of O_2_ is calculated to be 90.2%, which proves high sensitivity in ratiometric luminescence O_2_ detection.

Additionally, measurement of phosphorescence lifetime is another reliable method for quantitative oxygen sensing. By using eqn (3), lifetime-based analysis of oxygen content has also been realized. As seen in Fig. 2b, the emission lifetime at 704 nm changes from 1.10 μs to 0.11 μs in pure nitrogen or pure oxygen, respectively. The remarkable lifetime variation and the linear relationship between lifetime and O_2_ content shown in Fig. 2d ensure accuracy and sensitivity in lifetime-based oxygen detection.

### Ratiometric luminescence and lifetime imaging of oxygen in cells

**Fig. 3:**
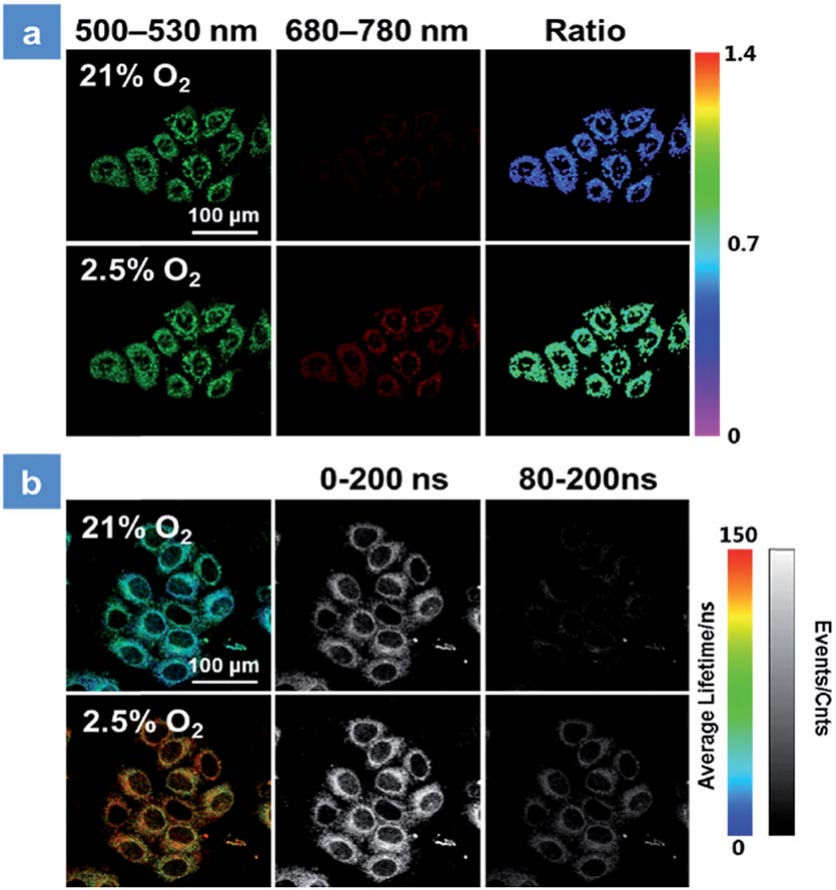
(a) Confocal laser scanning microscopy imaging of HeLa cells after incubation with **P1** at 2.5 and 21% oxygen contents. The green channels were acquired by collecting the luminescence signal from 500 to 530 nm, while the red channels were from 680 to 780 nm. The excitation wavelength was 488 nm. (b) Photoluminescence lifetime images (color images) and time-gated luminescence images (grey images) of HeLa cells. The lifetime was obtained by collecting the signal from 680 to 780 nm at 2.5 and 25% oxygen contents. 0 and 80 ns decay times were set for time-gated luminescence images. The scale bar is 100 μm. The concentration of the SPNs incubated in cells is 50 μg mL^–1^.

The good water-solubility and high oxygen-response sensitivity have indicated the promising potential of **P1** for mapping oxygen in biological systems. The cytotoxicity of **P1** is a vital factor to be concerned for biosensing and bioimaging. The cytotoxicity evaluation of **P1** was carried out based on the 3-(4,5-dimethyl-2-thiazolyl)-2,5-diphenyl-2*H*-tetrazolium bromide (MTT) assay. As shown in Fig. S3,† over 90% of the cells were viable after incubation with **P1** for 24 h in darkness, indicating the low cytotoxicity of **P1** in biological systems. Next, the biodegradability of **P1** was studied *in vitro* as well.3c,d,10e Myeloperoxidase (MPO) is a widely distributed peroxidase enzyme in immune cells. The reaction of MPO and H_2_O_2_ can generate hypochlorous acid, which can destroy proteins and even cell organelles. Upon adding with the mixture of MPO and H_2_O_2_, the absorption of **P1** remained the same for 48 h (Fig. S4†), indicating that **P1** could not be broken down by MPO. This result proves that **P1** is beneficial for the long-time monitoring of oxygen mapping. Before confocal laser scanning microscopy (CLSM) imaging, the cells were incubated with 50 μg mL^–1^**P1** for 4 h at the oxygen contents of 21% and 2.5%. In Fig. 3, CLSM images of cells were acquired under 488 nm irradiation. With the decrease of the oxygen level from 21% to 2.5%, the signal from BODIPY units (500–550 nm, green channel) remains unchanged, while that from iridium(iii) complex units (680–780 nm, red channel) shows a remarkable enhancement. Moreover, as shown in Fig. 3a, ratiometric luminescence oxygen sensing in cells has also been confirmed by the evident change of ratio values (*I*_704nm_/*I*_515nm_) of **P1**, which demonstrates its sensitive intracellular oxygen detection ability.^21^ Furthermore, the long emission lifetime of **P1** has been utilized for time-resolved luminescence imaging, which includes phosphorescence lifetime imaging (PLIM) and time-gated lifetime imaging (TGLI). According to PLIM images displayed in Fig. 3b, the lifetime of **P1** under normoxia conditions (70 ns) is shorter than that under hypoxia conditions (110 ns), which is consistent with the results in solution. Then, TGLI was conducted for highlighting the advantages of the long-lived phosphorescence of **P1** in Fig. 3b. After setting a time delay of 80 ns, the lifetime signal can still be observed clearly. Because of the oxygen induced quenching, the signal in hypoxia was stronger than that in normoxia. According to these results acquired *via* TGLI techniques, the signal-to-noise ratio in sensing intracellular O_2_ can be evidently elevated by eliminating the background interference in complicated environments.^22^

### **P1**-mediated PDT

Next, the PDT effect evaluation of **P1** was studied. To capture the ^1^O_2_ generated by **P1** in cells, 2,7-dichlorofluorescin diacetate (DCFH-DA) was chosen as an intracellular singlet oxygen indicator.^23^ After incubation with **P1** and DCFH-DA for 4 h, HeLa cells treated under different conditions were monitored by CLSM. The emission signal collected from DCF between 500 and 550 nm is shown in Fig. S5.† In contrast to the cells treated in darkness, those treated under xenon lamp irradiation display bright fluorescence of DCF, illustrating that the abundant ^1^O_2_ has been produced by **P1**. Before PDT, the photoinduced cytotoxicity of **P1** has been analyzed in HeLa cells *via* the MTT assay. As shown in Fig. S6,† the death cell population increases along with both extended minutes and increased dosage of SPNs, which confirms the efficient PDT of **P1**. For further demonstrating the PDT effect of **P1**, the annexin V-FITC/PI kit has been applied in this experiment for observing the apoptosis state of cells.^24^ As seen in the images of Fig. S7a and b,† the signal of annexin FTIC/PI cannot be observed in blank or dark groups, indicating that **P1** has low toxicity without light irradiation. Moreover, compared to the cells treated with the ROS cleaner *N*-acetyl-l-cysteine (NAC) (Fig. S7c†), the non-NAC-added group exhibits bright green fluorescence of DCF (Fig. S7d†), furtherly demonstrating that the destructive ^1^O_2_ has been generated for killing HeLa cells.

### Oxygen-related PDT evaluation *in vivo*

So far, the significantly hypoxic microenvironment has been demonstrated as a common feature inside solid tumors.^25^ Real-time O_2_ monitoring in the tumor environment can help to predict the PDT efficiency and give suggestions for subsequent treatment. Before *in vivo* experiments, the ^1^O_2_ yield influenced by O_2_ content is investigated firstly in solution. The mixture of **P1** and ABDA was bubbled with different contents of O_2_. After irradiation by a low-powered 475 ± 20 nm xenon lamp for 1 min, the absorbance of ABDA at 400 nm decreased, which indicated the increase in singlet oxygen concentration. The results of the ^1^O_2_ generation rate under different conditions have been presented in Fig. S8.† By using eqn (2), the light induced ^1^O_2_ yields at different O_2_ levels of 5, 10, 15 and 21% are calculated to be 20, 35, 65 and 97%, respectively. The positive relationship between the O_2_ content and ^1^O_2_ yield directly demonstrates that O_2_ plays a significant role in PDT.

**Fig. 4:**
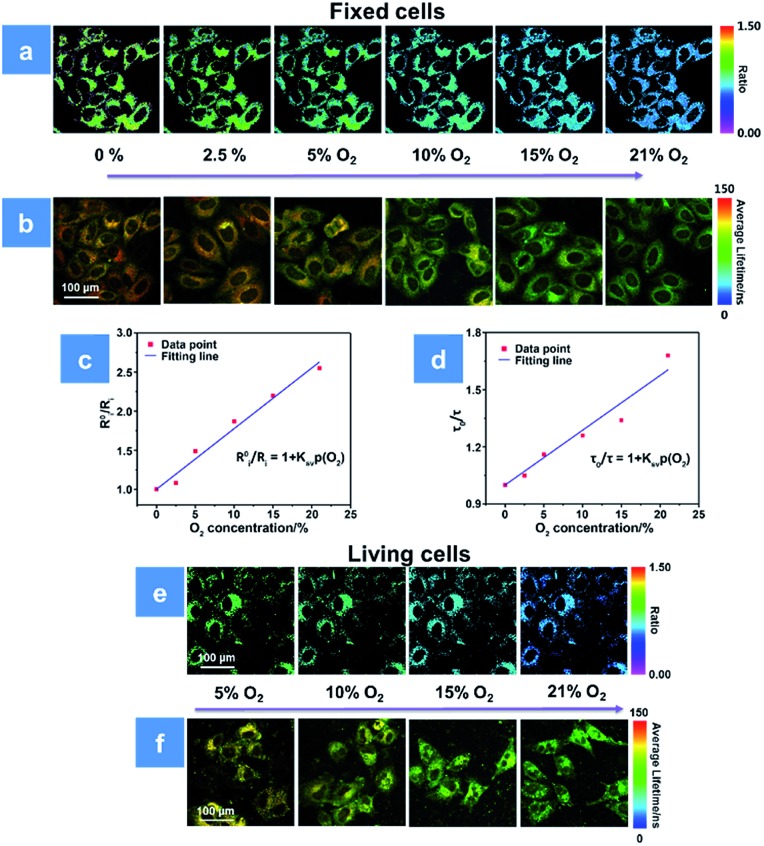
(a and b) Ratiometric luminescence and lifetime imaging of **P1**-incubated fixed HeLa cells at oxygen contents of 0, 2.5, 5, 10, 15 and 21%, respectively. (c) Stern–Volmer fitting curve between *R*0i*/R*_i_ and O_2_ content in fixed cells (*R*^2^ = 0.997, *K*_SV_ = 0.077%^–1^). (d) Stern–Volmer fitting curve between *τ*_0_/*τ* and O_2_ content in fixed cells (*R*^2^ = 0.987, *K*_SV_ = 0.028%^–1^). (e and f) Ratiometric and phosphorescence lifetime imaging of **P1**-incubated living HeLa cells at oxygen contents of 5, 10, 15 and 21%, respectively. The scale bar is 100 μm. The concentration of the SPNs incubated in cells is 50 μg mL^–1^.

For further evaluating the PDT process with **P1**, we combined the ratiometric luminescence and lifetime imaging techniques to determine the intracellular oxygen concentration of living cells. The fixed cells are selected for simulating the biological environment beforehand, because the O_2_ content is supposed to be the same inside and outside the fixed cells, which ensures the reliability of the results. As seen in Fig. 4a, after incubation with **P1** for 2 h, the fixed HeLa cells were cultured at the extracellular O_2_ contents of 0, 2.5, 5, 10, 15 and 21%. The intensity ratio (*I*_704 nm_/*I*_515 nm_) reveals a decreasing trend upon increasing O_2_ content, and the calibration curve has been fitted according to eqn (3). Then, the real O_2_ content in living cells is determined in terms of the calibration curve and shown in Fig. 4c. When the O_2_ content outside the living cells is 5%, **P1** in cells exhibits an intensity ratio of 0.87 and the real O_2_ content is calculated to be 4%. As the O_2_ content increases to 21%, the ratio value decreases to 0.51 and the real O_2_ content is calculated to be 16% (Fig. 4e). Photoluminescence lifetime imaging is another effective tool for real O_2_ content assessment. Similarly, as shown in Fig. 4b, the PLIM-based calibration curve has been fitted in terms of the luminescence lifetime distributions of **P1** in fixed cells at various O_2_ contents. And according to the calibration curve in Fig. 4d, the real O_2_ content in living cells has been calculated to be 5% and 21% at the extracellular O_2_ contents of 4% and 18%, respectively (Fig. 4f). We found that the real O_2_ concentration calculated by both ratiometric imaging and PLIM is lower than that outside the cells, which is caused by the oxygen consumption of cellular activity (Tables S2 and S3†). Thus, the ratiometric luminescence and lifetime imaging techniques provide high sensitivity and signal-to-noise ratio for real oxygen content detection in cells. Making full use of the real-time O_2_ imaging ability of **P1**, the subsequent PDT assessment can be realized.

The time series model of CLSM has been utilized to track and distinguish the apoptosis and death of cells after different treatments. As shown in Fig. S9 and S10,† the fluorescence of annexin V-FITC/PI cannot be observed in the blank or dark group during 4 h, which is in line with the above results shown in Fig. S8a and b.† In contrast, the fluorescence signal of annexin V-FITC appears obviously 1 h after irradiation, indicating the early apoptosis of HeLa cells. Then, the apoptotic signal collected from PI dyes in the cell nucleus can be observed 2 h after irradiation (Fig. S11†), demonstrating the death process of HeLa cells. By contrast, the cell-killing ability becomes less effective due to the lack of oxygen as the ^1^O_2_ source (Fig. S12†). Therefore, the highly efficient PDT of **P1** has been confirmed by qualitative CLSM analysis. For better understanding the PDT ability of **P1**, the quantitative analysis of **P1**-induced apoptosis has been studied *via* flow cytometry analysis (Fig. S13†). The population statistics of apoptosis cells treated with **P1**-induced PDT is determined to be 58.5% (Fig. S13d†). In contrast, just a few cells died in the control group (Fig. S13a–c†). In addition, the proportion of apoptotic HeLa cells increases as the exposure time extends (Fig. S13e–h†), which is in line with the MTT results shown in Fig. S6b.† More importantly, the oxygen-dependent apoptotic effect is also investigated (Fig. S13i–l†). A notable growth in the number of apoptotic cells is observed with increasing O_2_ content. These results point out that the content of O_2_ greatly impacts the PDT efficiency and provides effective instructions for *in vivo* experiments.

### Anticancer studies in xenograft zebrafish

**Fig. 5:**
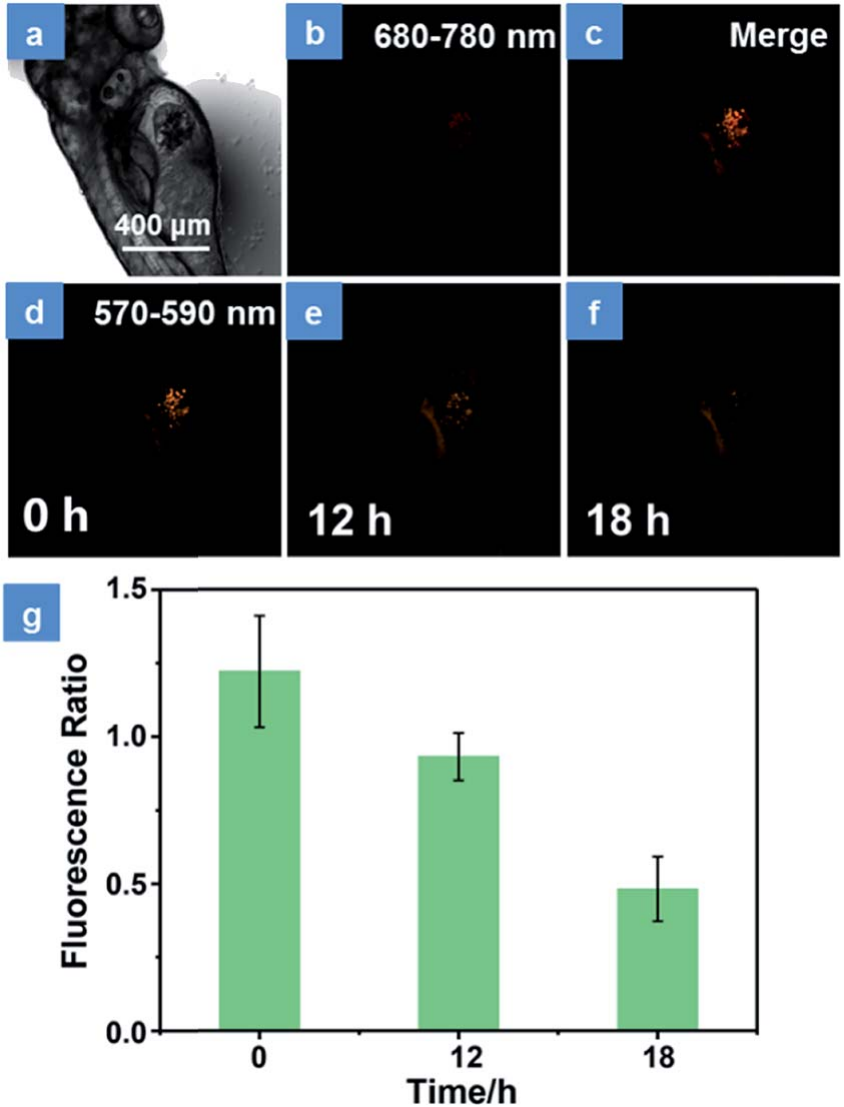
Representative confocal laser scanning microscopy images of xenograft zebrafish with the treatment of **P1**. (a) Bright field of zebrafish. (b) The emission signal of **P1** in zebrafish. (c) Merge of the emission signal of **P1** and fluorescent protein in zebrafish. (d–f) The emission of fluorescent protein collected in zebrafish after injection of **P1** for 0 h, 12 h and 18 h, respectively. (g) The histogram of intensity ratio between 570 and 590 nm to that between 680 and 780 nm. The emission of **P1** was collected between 680 and 780 nm and the emission of fluorescent protein was collected between 570 and 590 nm. Error bars indicate SD (*n* = 3). *P* < 0.05 (two-tailed Student's *t*-test). The scale bar is 400 μm. The concentration of the SPNs injected into the zebrafish is 100 μg mL^–1^. PDT was conducted with a xenon lamp (475 nm, 30 mW cm^–1^) for 15 min.

Encouraged by the excellent PDT performance of **P1***in vitro*, the *in vivo* treatment has been assessed using HeLa cells xenograft zebrafish. Firstly, the PDT effect in transfected HeLa cells was studied. As shown in Fig. S14,† the HeLa cells have been transfected with the DsRed fluorescent protein expression vector according to operating instructions. In order to demonstrate the photostability of fluorescent protein during the PDT process, the cells were exposed to a 475 nm xenon lamp for 15 min. The emission of fluorescent protein observed using an inverted fluorescence microscope displays no significant decline (Fig. S14a†), which is proved to have good photostability in the PDT process. After incubation with **P1** for 4 h, the cells were divided into two groups. There is no obvious difference in the population of cells between the control group (Fig. S14b†) and the dark group (Fig. S14c†), revealing the low dark toxicity of **P1**. In contrast, under irradiation by a xenon lamp for 15 min, the emission of fluorescent protein decreased 4 h later (Fig. S14d†). This is because the high levels of ^1^O_2_ induced by **P1** can damage the DNA, cleave the protein and then give rise to the cell apoptosis and necrosis.^26^ These results suggest that **P1** is an effective tool for cell inhibition. For the purpose of simulating a cancer cell growth environment, the zebrafish xenograft model has been used to evaluate the PDT effects of **P1**. HeLa cells were firstly xenotransplanted into the yolk sac of 3 day zebrafish for 12 h and then treated with **P1** for 4 h *via in situ* injection inside the cell cluster before CLSM imaging. The emission intensity of fluorescence protein in cancer cells was monitored for assessing the cell survival state. As expected, the control group displays almost no intensity change of cancer fluorescence, which indicates good biocompatibility and low toxicity in darkness (Fig. S15†). Then, representative images of xenotransplanted zebrafish injected with **P1** (100 μg mL^–1^) and exposed under 15 min irradiation by a xenon lamp are depicted in Fig. 5. The NIR phosphorescence emission (680–780 nm) of **P1** has a good overlap with the cancer fluorescence (570–590 nm) (Fig. 5b–d), which guarantees the maximized PDT effect for cancer cells. Consistent with the results *in vitro*, the number of emissive cells significantly decreased during 18 h (Fig. 5d-f). The reduced fluorescence intensity of cancer cells has been recorded in Fig. 5g. As the time increased, the cancer cells gradually died and the ratio of the intensity in cancer cells between 570 and 590 nm to that between 680 and 780 nm decreased, which proves the excellent cancer inhibition ability of **P1** in zebrafish.

### Anticancer studies in mice models

**Fig. 6:**
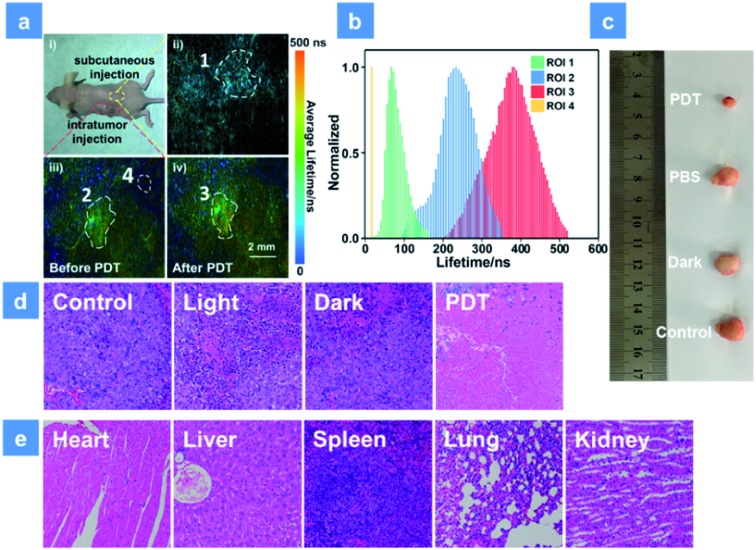
(a) Phosphorescence lifetime imaging of **P1** excited at 515 nm in a tumor-bearing mouse. (a_i_) Photo of the tumor xenografted mice after subcutaneous and intratumor injection with **P1**. (a_ii_) Phosphorescence lifetime imaging of the subcutaneously injected area (ROI 1). (a_iii_) Phosphorescence lifetime imaging of the intratumorly injected area before irradiation (ROI 2) and the background fluorescence area (ROI 4). (a_iv_) Phosphorescence lifetime imaging of the intratumorly injected area after irradiation (ROI 3). All the ROI areas were circled with white dotted lines and the excitation wavelength was 515 nm. The scale bar is 2 mm. (b) Lifetime distributions of **P1** in different regions of the mouse. A 600 nm long pass filter was used for collecting the lifetime signal of **P1**. (c) Photos of tumor tissue excised from mice in different groups after 14 d treatment. (d) H&E analysis of tumor tissue from different groups after two weeks treatment. (e) H&E analysis of mice normal organs in the PDT group after two weeks therapy. The concentration of the SPNs injected into the mouse is 3 mg kg^–1^. PDT was conducted using a xenon lamp (200 mW cm^–2^, 15 min).

For further simulating a human body environment, PDT in the tumor-bearing mice has been studied. As shown in Fig. 6a, 3 mg kg^–1^ SPNs were injected beneath the skin (yellow area) or into the tumor tissue (red area). Then, the lifetime distribution curves of **P1** were acquired by using the *in vivo* PLIM system (Fig. 6b). The lifetime of **P1** in ROI 1 was about 68 ns. In contrast, the lifetime lengthened to be about 233 ns in ROI 2 of the tumor, which was caused by the hypoxia feature of tumor tissues. Moreover, after 15 min irradiation by a xenon lamp, the phosphorescence lifetime of ROI 3 became 380 ns and was longer than that without treatment (ROI 2). This result suggests that the sensitive O_2_ detection ability of **P1** is beneficial for real-time monitoring of O_2_ consumption during PDT. In addition, the lifetime collected from the background area (ROI 4) was only 20 ns and could be obviously distinguished from the signal of **P1**. This suggests that the high signal to noise ratio in O_2_ mapping utilizing **P1** can provide a reliable visual feedback during PDT treatment. Then, PDT in tumor-bearing mice has been studied. To distinguish the roles played by the light source, oxygen and photosensitizer, all mice were randomly assigned to four groups (Fig. S16†). The tumor volume and mice body weight were continuously recorded every two days and used to evaluate the treatment effect for two weeks (Fig. S17a and b†). The PBS-injected group shows an approximately 8-fold larger tumor volume than the initial state and the only xenon lamp irradiated group (475 nm, 200 mW cm^–2^, 15 min) exhibits a similar tumor growth rate, suggesting that the light irradiation has no evident impact on tumor growth. Then, **P1** was intratumorally injected into the tumor tissue followed by different treatments after 2 h injection in PDT and dark groups. **P1**-treated mice in the dark group exhibit a similar tumor growth to the control group owing to the low photocytotoxicity of **P1**. Then, the excellent tumor inhibition ability of **P1** is found in the PDT group under 475 nm irradiation, and the experimental photographs of tumors excised from representative mice visually reveal the tumor size after treatment with **P1** (Fig. 6c). These results confirm the excellent PDT effect for killing tumors induced by **P1**. Histology results of the mice injected with **P1** have been studied as well (Fig. 6d). By utilizing the hematoxylin and eosin (H&E) staining method, tumor tissue necrosis is clearly observed. Compared with other control groups, a high degree of tumor cell necrosis and apoptosis is found in the PDT group, evidencing tumor cell death after **P1**-mediated PDT. For the purpose of evaluating the *in vivo* biosafety of **P1**, the body weight of mice in different groups was recorded and the metabolic time of **P1** in solid tumors was studied using small animal *in vivo* imaging systems (Fig. S17b and c†). After **P1** was injected into the tumor intratumorally, the phosphorescence signal of **P1** (680 ± 20 nm) was collected. The maximum intensity of **P1** can be observed in tumor tissues 2 h after the injection. Within 36 h, the signal gradually decreased, demonstrating that **P1** could also be excreted rapidly from the tumor. In addition, the body weight of mice in all groups slightly fluctuates after 14 days treatment, indicating that **P1** has no significant side effects on healthy mice (Fig. S17b†). Moreover, the histological examination of primary organs excised from **P1**-injected nude mice after 14 days PDT treatment has been performed by H&E as well. The photos of the stained mice organs were observed under microscopy (Fig. 6e). After two weeks PDT, no obvious necrosis or inflammatory response happened to the examined mice organs, confirming the favorable biocompatibility of **P1***in vivo*.

## Conclusions

In summary, we have successfully utilized the FRET strategy to design dual-emissive semiconducting polymer nanoparticle-based photosensitizers containing BODIPY derivatives and near-infrared phosphorescent iridium(iii) complexes for amplified ^1^O_2_ generation in PDT. In the SPNs, the BODIPY units served as the energy donors in the FRET process and enhanced the light absorption ability of the SPNs. The NIR emissive iridium(iii) complexes were employed as the energy acceptors and photosensitizers, which have been demonstrated to provide the SPNs with high ^1^O_2_ quantum yield. In the meantime, the ionized semiconducting polymer can easily self-assemble to form nanoparticles homogeneously in water and the conjugated backbone has been demonstrated to offer effective shielding for the two luminophores from photobleaching. Due to the rational structural design, together with the FRET-based synergistic effect of the two luminophores in SPNs, a high ^1^O_2_ quantum yield (0.97) has been successfully achieved, which is among the best reported for PSs. In addition, taking advantage of the balanced process between the O_2_-related photophysical variation (PL intensity ratio or lifetime value) and ^1^O_2_ production, **P1** has also been used as a multifunctional theranostic platform for image-guided PDT treatment *in vitro* and *in vivo*. All the results reveal that the SPNs integrating and amplifying the advantages of all the components successfully achieve high PDT efficiency as well as excellent O_2_ imaging capability. We believe that these results would inspire the development of more excellent SPN-based photosensitizers for cancer theranostics.

## Experimental section

The detailed information of materials, instruments, synthesis and characterization of SPNs, spectral tests, cell culture, cell xenotransplantation, *in vitro*/*in vivo* imaging and PDT experiments can be found in the ESI.†

## Animal models

All the nude mice and zebrafish were bought from the Comparative Medicine Centre of Yangzhou University and Model Animal Research Center of Nanjing University, respectively. All the animal experiments were conducted in line with the specifications of The National Regulation of China for Care and Use of Laboratory Animals and approved by the Jiangsu Administration of Experimental Animals.

## Conflicts of interest

The authors declare no competing financial interest.

## Supplementary Material

Supplementary information
