# Supplementary material for: Enhancing singlet oxygen generation in semiconducting polymer nanoparticles through fluorescence resonance energy transfer for tumor treatment
Source: Chem Sci. 2019 Apr 11;10(19):5085–94. doi: 10.1039/c8sc05501g (PMC6524665; doi:10.1039/c8sc05501g)
Supplement: Supplementary file 1 [file SC-010-C8SC05501G-s001.pdf]

Electronic Supplementary Material (ESI) for Chemical Science.
This journal is © The Royal Society of Chemistry 2019

Electronic Supplementary Information

Enhancing singlet oxygen generation in semiconducting polymer nanoparticles through fluorescence resonance energy transfer for tumor treatment

Jiayang Jiang,[‡a] Yuanyuan Qian,[‡a] Zihan Xu,[a] Zhuang Lv,[a] Peng Tao,[a] Mingjuan Xie,[a] Shujuan Liu,[a] Wei Huang,*[ab] and Qiang Zhao*[a]

[a]Key Laboratory for Organic Electronics and Information Displays, Institute of Advanced Materials (IAM), Jiangsu National Synergetic Innovation Center for Advanced Materials (SICAM), Nanjing University of Posts and Telecommunications (NUPT), Nanjing 210023, P. R. China. E-mail: iamsjliu@njupt.edu.cn; iamqzhao@njupt.edu.cn.

[b]Shaanxi Institute of Flexible Electronics (SIFE), Northwestern Polytechnical University (NPU), Xi'an 710072, Shaanxi, China. E-mail: provost@nwpu.edu.cn.

‡ J. Y. Jiang and Y. Y. Qian contributed equally to this work.

Experimental section

Materials: All the experimental materials and reagents were purchased from commercial suppliers and used without further purification except the other noted. All solvents were purified and dried according to standard procedures before used. All aqueous solutions were prepared with deionized water. Cell culture flasks, culture dishes and confocal petri dishes were bought from Thermo Fisher. Discosoma sp. red fluorescent protein (DsRed) expression vector pCMV-C-DsRed were purchased from Biyotime. Lipofectamine® 2000 reagent was acquired from Thermo Fisher. Dulbecco's modified eagle medium (DMEM) and fetal bovine serum (FBS) were purchased from Gibcco.

Instruments: The NMR spectra were measured by Bruker Ultra Shield Plus 400 MHz NMR instrument using deuterated solvents at 298 K referenced against external Me_4Si. Chemical shifts were shown in parts per million. The mass spectra were recorded on Bruker autoflex matrix-assisted laser desorption ionization time-of-flight (MALDI-TOF) mass spectrometer. The weight-average molecular mass (M_w) and number-average molecular mass (M_n) of the semiconducting polymers (dissolved in THF) were characterized by gel permeation chromatography via a calibration curve of polystyrene standard at 308 K. Transmission electron microscopy (TEM) pictures were obtained on a JEOL JEM-2100 transmission electron microscope at 100 kV acceleration voltage. The average particle size at room temperature was obtained by dynamic light scattering (DLS) on Zetasizer Nanoseries (Nano ZS90). The UV-visible absorption spectra were acquired with a Shimadzu UV-3600 spectrophotometer. The excitation and emission spectra were carried out by using Edinburgh FL 920 spectrophotometer. An integrating sphere was employed to determine the absolute quantum yields of the compounds in N_2 atmosphere. Excited-state lifetime studies were performed using a semiconductor laser unit on Olympus Edinburgh LFS-920 spectrometer. The luminescence decay profile data were analyzed via a software package provided by Edinburgh Instruments. The time-resolved luminescence imaging experiments of cells were carried out on an Olympus FV1000 laser scanning confocal microscope and the lifetime data was

calculated by correlative and professional software provided by Becker & Hickl GmbH Company. The lifetime imaging experiments of tumor bearing mice were performed by using a Becker & Hickl GmbH DCS-120 PLIM system. The flow cytometer analysis was conducted on the FlowSight imaging flow cytometer manufactured by Merck Millipore cooperation. The zebrafish microinjection system was established by Eppendorf. The luminescence imaging of mice was carried out on IVIS Lumina K Series III In Vivo Imaging system manufactured by PerkinElmer company. Photographs of tumor bearing mice were taken with a camera phone.

Cell culture: The Hela cell lines were purchased from Institute of Biochemistry and Cell Biology, SIBS, CAS (China). The cells were cultured with DMEM and provided with 10% fetal bovine serum at 37 ^{o}C under 5% CO_2.

Preparation of transfection cells: Hela cells were transfected with the red fluorescent protein expression vector pCMV-C-DsRed (Biyotime, China) using Lipofectamine® 2000 reagent (Thermo Fisher) according to the manufacturer's instructions. After 36 h transfection, the cells which stably expressed the fluorescence protein (λ_{ex} = 559 nm, λ_{em} = 570–600 nm) were selected for the subsequent experiments.

Hela cell xenotransplantation: The zebrafish was acquired from Model Animal Research Center of Nanjing University. All the animal experiments were conducted in line with the specifications of The National Regulation of China for Care and Use of Laboratory Animals and approved by the Jiangsu Administration of Experimental Animals. Before xenotransplantation, two-days-old zebrafish were anesthetized with 2-phenoxyethanol solution and lay on prepared sepharose gel. Transfected Hela cells were suspended in 20 ml of Hanks' balanced salt solution. The avascular region of the yolk sac was then injected with 200−300 cells using the glass needles and the injection system (Eppendorf).

Cell viability assessment: The *in vitro* cytotoxicity of **P1** toward Hela cells was evaluated by the methyl thiazolyl tetrazolium (MTT) based assay. Briefly, cells in exponential growth period were seeded into 96-well cell culture plate at proper

concentration. And then **P1** was added with the concentrations of 0, 10, 20, 40 and 50 μg/mL. After cultured for 24 h at 37 °C under 5% CO_2, cells in each well were added with MTT (5 mg/mL, dissolved in PBS solution) and incubated for another 4 h. While in the phototoxicity test, the cells were exposed under xenon lamp (475 nm, 30 mW/cm^2) before another 4 h incubation. Then the culture solution was removed and 150 μL DMSO was added into each well. After shaken for 10 min, an enzyme-linked immunosorbent assay reader was used to measure the optical density (OD value) of each well (monitored at 570 nm). The following formula was used to calculate the viability of cells:

Viability (%) = (mean of OD value of treatment group/mean of OD value of control group) × 100.

Hypoxia atmosphere control: For carrying out the imaging or PDT experiments under a certain oxygen content (2.5, 5, 10, 15 and 21%), the live cell station equipped with a flowmeter was used as the function of controlling the flow rates of O_2 and N_2. The subsequent experiments were carried out after the cells were incubated under certain oxygen content for 30 min.

ROS detection in Hela cells: Hela cells were incubated with 50 μg/mL **P1** for 4 h and treated with DCFH-DA (10 μM) for 15 min at 37 °C. After irradiated by xenon lamp (475 nm, 30 mW/cm^2) for 10 min, the fluorescence intensity of DCF was detected with the confocal microscopy by collecting signal between 500 and 550 nm (λ_{ex} = 488 nm).

Annexin V-FITC/PI assay: Hela cells were incubated with 50 μg/mL **P1** in normoxia for 4 h and then the cells were irradiated by xenon lamp (475 nm, 30 mW/cm^2) for 10 min (when studying the apoptosis in hypoxia, the cells should be cultured for another 1 h under 5% O_2 before irridiation). Then the cells were stained with Annexin V-FITC and PI according to the agent instruction (KeyGEN, China). Confocal microscopy and flow cytometry were used to collect the fluorescence intensity from the cells (Annexin V-FITC, λ_{ex} = 488 nm, λ_{em} = 500–560 nm; PI, λ_{ex} = 488 nm, λ_{em} = 600–680 nm).

Luminescence and lifetime imaging of Hela cell: The imaging experiments were carried out with an Olympus FV1000 laser scanning confocal microscope equipped with a temperature controller and a 40 × objective lens. Hela cells growing in log phase were seeded into confocal plate at proper concentration until their adherence. **P1** was added to the confocal plate with the concentration of 50 μg/mL and then the cells were incubated for 4 h at 37 °C under 5% CO_2. After washed with PBS, **P1** in Hela cells was excited at 488 nm by a semiconductor laser equipped on the confocal microscope. The luminescence signals from **P1** were collected at 500–550 nm and 680–780 nm by charge coupled device module and the lifetime signal was collected by a 600 nm long pass filter and handled by time-correlated single photon counting module.

PDT of zebrafish: The imaging experiments of zebrafish were carried out with an Olympus FV1000 laser scanning confocal microscope equipped with a temperature controller and a 10 × objective lens. After one day xenotransplantation of transfected Hela cells, 100 μg/mL **P1** were injected into the Hela clusters of the zebrafish. The injected zebrafish were allowed to be cultured for another 4 h and then irradiated by xenon lamp (475 nm, 30 mW/cm^2) for 15 min. The treated zebrafish were finally put under the confocal microscopy for collecting the luminescence signals. The signal from transfected Hela cells was collected between 570–590 nm and that from **P1** was collected between 680–780 nm (λ_{ex} = 488 nm).

Tumor model: Six-weeks-old female nude mice were bought from Center for Comparative Medicine Centre of Yangzhou University and all the experiments were conducted in line with the specifications of The National Regulation of China for Care and Use of Laboratory Animals and approved by the Jiangsu Administration of Experimental Animals. The Hela tumor models were successfully established by subcutaneous injection into the left back of each mouse with 150 million Hela cells suspended in 150 μL PBS. The mice were treated under different conditions when the tumor volumes grew at approximately 50 mm^3.

Lifetime imaging of tumor-bearing mice: The solution of **P1** (3 mg/kg) was directly injected inside the solid tumor on mice. The mice were anaesthetized with isoflurane

gas for 15 min and then taped on the imaging platform for PLIM imaging. The phosphorescence signal was collected with a 600 nm long pass filter by a DCS-120 scanner (λ_{ex} = 515 nm) and the data were analyzed by Becker & Hickl GmbH software package.

***In vivo* antitumor studies**: All mice were randomly distributed into four groups (four mice each group): 1) control group (only injected with PBS); 2) dark group (injected with **P1** and treated in darkness); 3) light irradiation group (only treated with light); 4) PDT group (injected with **P1** and exposed to light). When the tumors reached the volume of approximately 50 mm^3, **P1** was injected intratumorally into the tumor bearing mice at a dose of 3 mg/kg. Every two days, PDT was conducted by a xenon lamp (200 mW/cm^2, 15 min) and the relative tumor volumes and body weights were measured. After 14 days treatment, the mice were sacrificed in terms of the institutional guidelines. Tumors and organs were obtained and then fixed. The therapeutic results of each group were evaluated by measuring the tumor volumes after 14 days (tumor volume (V) = $l \times w^2/2$, w is the width of tumor and l is the length). V/V_0 was recorded to figure out the relative tumor volume (V_0 is the corresponding tumor volume when the treatment is initiated).

Statistical Analysis: The luminescence data were provided in the form of mean ± standard deviation, and statistical comparisons between two groups were conducted by t-test. Statistical significance was considered to be at $P < 0.05$.

Synthesis

Benzothiophen-2-ylboronic acid, 1-chloroisoquinoline, 4-methylbenzaldehyde, 2,4-dimethyl-1H-pyrrole and $IrCl_3 \cdot 3H_2O$ were purchased via commercial channels and used without further purification.

1-Benzothienyl-2-isoquinoline (**btq**, **5**): 1-Benzothiophen-2-ylboronic acid (1.50 g, 8.43 mmol) was added to a mixture of 1-chloroisoquinoline (1.37 g, 8.40 mmol) toluene (30 mL), ethanol (10 mL), and 2 M K_2CO_3 aqueous solution (10 mL) with vigorous stirring at 85 °C for 24 h under N_2 atmosphere. After cooling down, the solution was washed with water and dichloromethane. The organic layer was collected and then purified by silica gel chromatograph using petroleum ether/ dichloromethane (1:1, v/v) to obtain the white powder. Yield: 83 %. ^1H NMR (400 MHz, $CDCl_3$): δ (ppm): 8.62 (s, 1H), 8.61 (s, 1H), 7.91 (m, 3H), 7.85 (s, 1H), 7.74 (t, J = 7.6 Hz, 1H), 7.66 (m, 2H), 7.41 (m, 2H).

$[Ir(btq)_2Cl]_2$ (**6**): $IrCl_3 \cdot 3H_2O$ (1.0 g, 2.83 mmol) and **3** (1.63 g, 6.23 mmol) were heated in a mixture of 2-ethoxyethanol and water (40 mL, 3:1 v/v) under N_2 atmosphere at 110 °C for 24 h. After cooling to room temperature, the precipitate was filtered off and washed with water and ethanol to give the crude marroon solid.

1,3-Bis(4-bromophenyl)propane-1,3-dione (**7**): This compound was prepared according to the literature procedure.[1]

$[Ir(btq)_2(BrdbmBr)]^+(PF_6)^-$ (**1**): A mixture of cyclometalated iridium(III) chloro-bridged dimer $[Ir(btq)_2Cl]_2$ (1.00 g, 0.67 mmol) and 1,3-bis(4-bromophenyl)propane-1,3-dione (0.56 g, 1.47 mmol) in solution of dichloromethane/methanol (1:1 v/v) was heated at 50 °C under nitrogen for 6 h and excess KPF_6 (1.35 g, 7.35 mmol) was added to the solution. The mixture was then cooled to room temperature, washed with water and dichloromethane. After the organic layer was collected and evaporated, dark brown solid was separated from the mixture by silica gel chromatograph using petroleum ether/dichloromethane (1:2, v/v). Yield: 21 %. ^1H NMR (400 MHz, $CDCl_3$) δ (ppm): 9.06 (d, J = 8.4 Hz, 2H), 8.31 (d, J = 6.4 Hz, 2H), 7.87 (d, J = 8.4 Hz, 2H), 7.76 (m,

6H), 7.57 (d, J = 8.4 Hz, 4H), 7.35 (d, J = 8.4 Hz, 4H), 7.24 (s, 1H), 7.11 (t, J = 6.8 Hz, 2H), 6.99 (s, 1H), 6.73 (t, J = 7.6 Hz, 2H), 6.47 (s, 1H), 6.403 (d, J = 8.0 Hz, 2H), 5.35 (t, J = 4.4 Hz, 1H). MALDI-TOF-MS m/z: 1093.59.
Because of the poor solubility of **1,** the ^{13}C NMR spectra are not present here.[2]

Monomer **2**, monomer **3** and monomer **4** were prepared according to the previous report.[3-5]

P1, **P2** and **P3** were prepared in the same way. The synthesis method of **P1** is present as an example.

Preparation of P1: Iridium(III) complex (**1**), BODIPY derivative (**2**) and 2,7-Dibromo-9,9-bis(2-(2-(2-bromoethoxy)ethoxy)ethyl)-9H-fluorene (**3**) were used for synthesizing the polymer by palladium-catalyzed Suzuki coupling reaction. A mixture of **1** (109.4 mg, 0.100 mmol), **2** (14.8 mg, 0.025 mmol), **3** (267.8 mg, 0.375 mmol), **4** (404.0 mg, 0.500 mmol), $Pd(PPh_3)_4$ (3.000 mol%) and small amount of phase-transfer catalyst tetrabutylammonium bromide were added into a 25 mL reaction flask. Then, 8 mL of 2 M Na_2CO_3 aqueous solution, 12 mL of THF were poured in the flask. The flask was vacuumed and refilled with N_2 three times. Then, the reaction was heated to 70 ºC and stirred under N_2 atmosphere. After 48 h the whole mixture was poured into 100 mL of MeOH. The coarse **P1** precursor was acquired by filtration and then purified with acetone by Soxhlet extraction to remove the small molecules (yield 37%). Quaternization of **P1** precursor was conducted with trimethylamine in THF solution for 24 h. After filtration, the precipitate was washed with acetone and dried for 24 h to obtain **P1**. Yield: 81%.

P1: ^1H NMR (400 MHz, $(CD_3)_2SO$): δ (ppm) 8.98 (br, Ar-H of Ir complex), 8.38 (br, Ar-H of Ir complex), 8.12–7.92 (br, Ar-H of Ir complex), 7.93 (m, Ar-H of fluorene, Ir complex), 7.73 (br, Ar-H of BODIPY, fluorene), 7.19 (br, Ar-H of Ir complex), 6.79 (br, Ar-H of Ir complex), 6.31 (br, Ar-H of BODIPY), 3.69 (br, side chain of fluorene), 3.20 (br, side chain of fluorene), 3.05−2.95 (m, side chain of fluorene), 2.73 (br, side chain of fluorene), 2.13 (m, side chain of fluorene).

P2: ^{1}H NMR (400 MHz, $(CD_3)_2SO$): δ (ppm) 8.07−7.99 (br, Ar-H of fluorene), 7.87−7.79 (br, Ar-H of fluorene, BODIPY), 7.68−7.38 (br, Ar-H of fluorene, BODIPY), 5.69 (s, Ar-H of BODIPY), 3.52−3.35 (br, side chain of fluorene), 3.25 (br, side chain of fluorene), 3.00 (br, side chain of fluorene).

P3: ^{1}H NMR (400 MHz, $(CD_3)_2SO$): δ (ppm) 8.99 (br, Ar-H of Ir complex), 8.74 (br, Ar-H of Ir complex), 8.38 (br, Ar-H of Ir complex), 8.14−7.92 (br, Ar-H of fluorine, Ir complex), 7.73 (br, Ar-H of Ir complex), 7.48 (d, Ar-H of Ir complex), 7.18 (br, Ar-H of Ir complex), 6.78 (br, Ar-H of Ir complex), 6.32 (br, Ar-H of Ir complex), 3.20 (br, side chain of fluorene), 3.05−2.95 (m, side chain of fluorene), 2.78−2.66 (br, side chain of fluorene), 2.31 (m, side chain of fluorene).

Preparation of SNPs: The method of reprecipitation by utilizing the amphipathicity of **P1** was conducted. First, the polymer **P1** was dissolved into the methanol solution. Then the solution was injected quickly into the deionized water and set under sonication for 5 min. The methanol was removed by N_2-blowing, and the nanoparticles were acquired by filtration through a 0.22 μm filter.

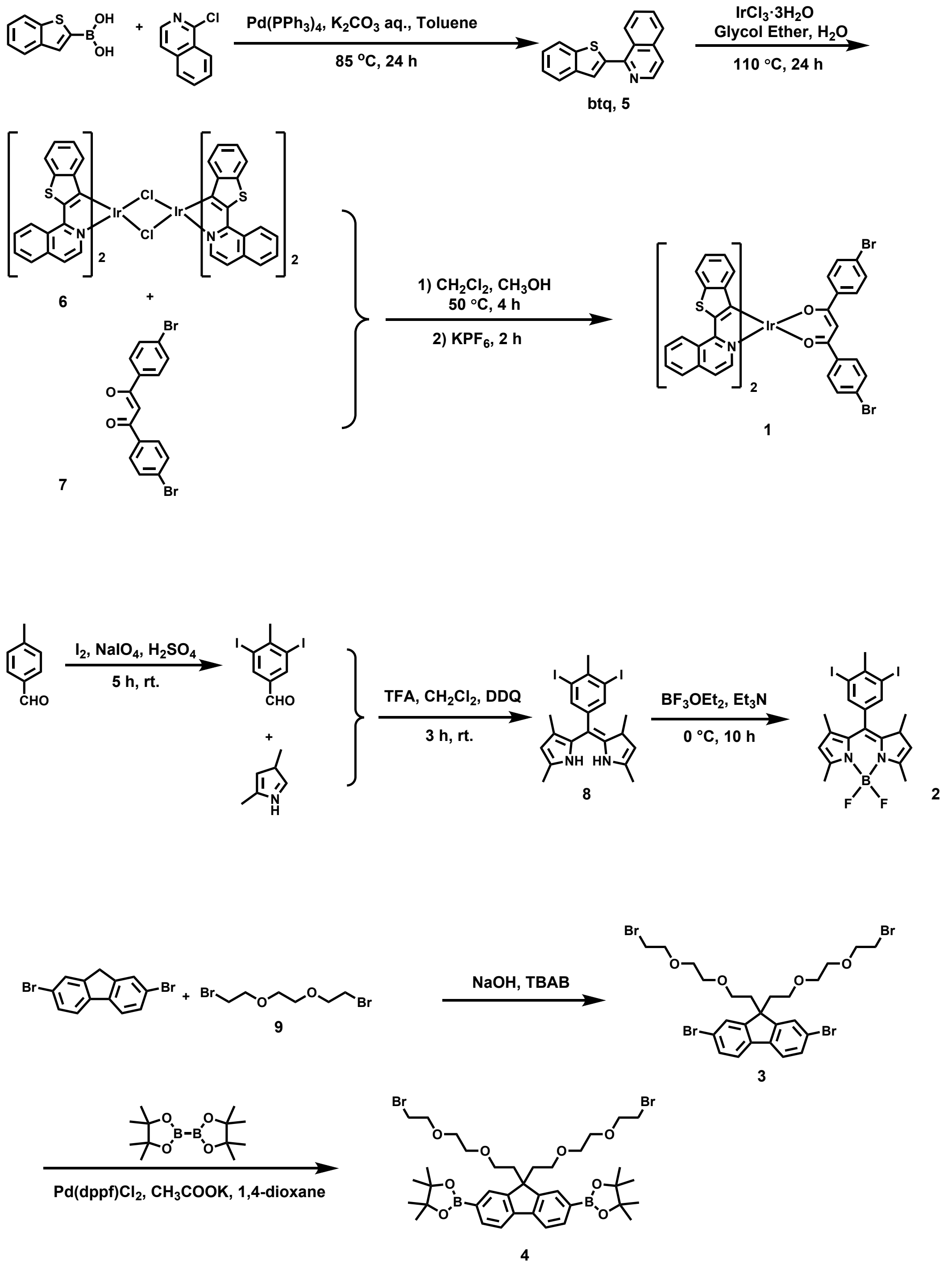

Scheme S1. Synthetic procedure of monomers.

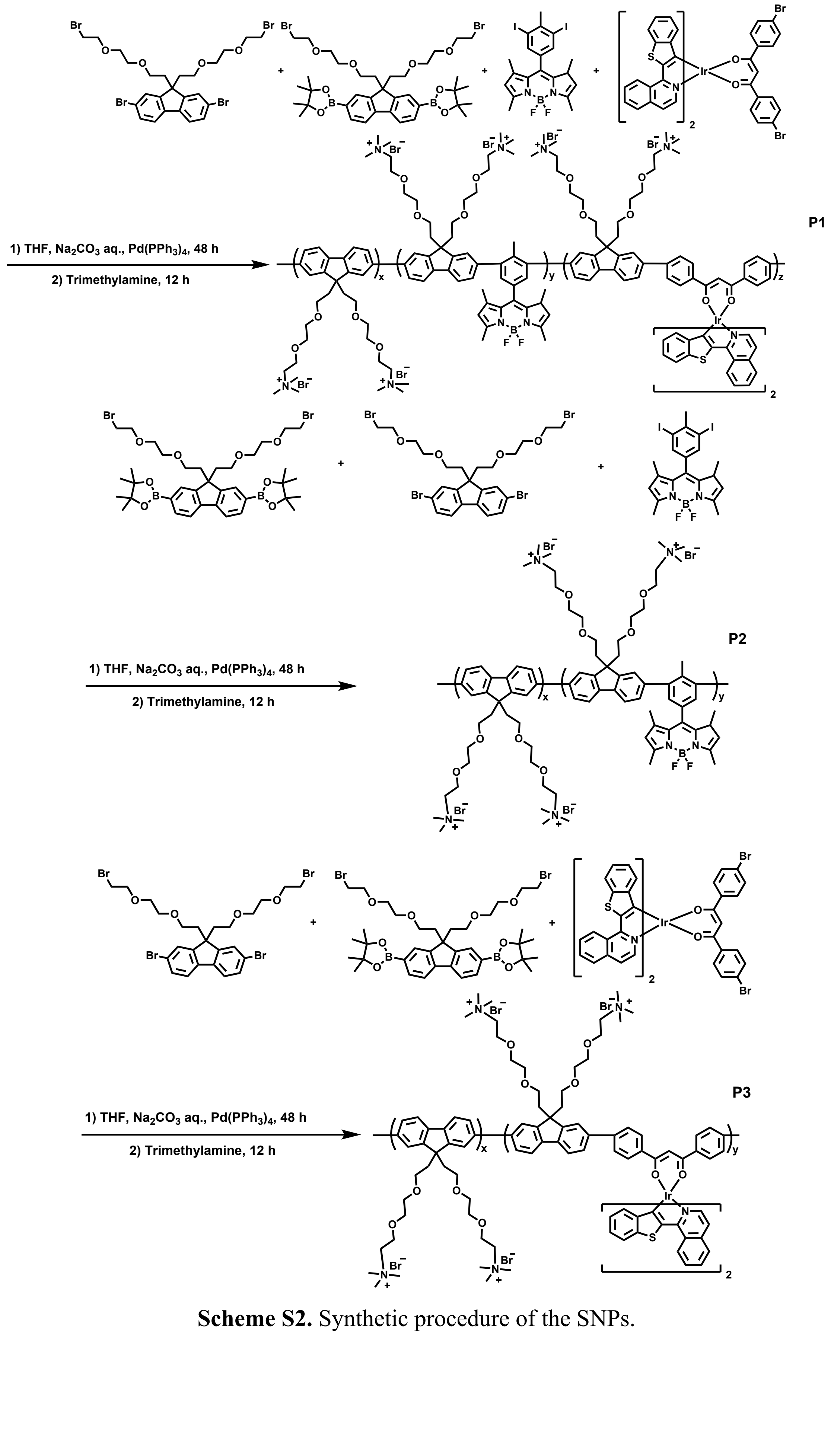

Scheme S2. Synthetic procedure of the SNPs.

Reference

1. H. F. Shi, S. J. Liu, H. B. Sun, W. J. Xu, Z. F. An, J. Chen, S. Sun, X. M. Lu, Q. Zhao and W. Huang, *Chem. Eur. J.*, 2010, **16**, 12158-12167.
2. S. Kesarkar, W. Mroz, M. Penconi, M. Pasini, S. Destri, M. Cazzaniga, D. Ceresoli, P. R. Mussini, C. Baldoli, U. Giovanella and A. Bossi, *Angew. Chem. Int. Ed.*, 2016, **55**, 2714-2718.
3. Y. Rong, C. Wu, J. Yu, X. Zhang, F. Ye, M. Zeigler, M. E. Gallina, I. Wu, Y. Zhang and Y. H. Chan, *ACS Nano*, 2013, **7**, 376-384.
4. Y. Du, Y. Wu, A. H. Liu and L. N. He, *J. Org. Chem.*, 2008, **73**, 4709-4712.
5. H. Shi, X. Ma, Q. Zhao, B. Liu, Q. Qu, Z. An, Y. Zhao and W. Huang, *Adv. Funct. Mater.*, 2014, **24**, 4823-4830.

Table S1. Molecular weight, polydispersity index, composition and photophysical properties of the SPNs.

				Monomer content/mol%					
	**M_w[a)]**	**M_n[b)]**	**PDI**	**feed ratio**	**actual ratio[c)]**	**τ[d)]/ns**	**τ[e)]/ns**	**QY[f)]**	**QY[g)]**
**P1**	27300	21747	1.26	20 : 5	10.1 : 4.8	0.99	447.42	0.37	0.11
**P2**	11750	19665	1.67	5	4.3	1.52	-	0.40	-
**P3**	15490	16970	1.10	20	14.7	-	404.14	-	0.13

a,b) Weight-average molecular mass (M_w) and number-average molecular mass (M_n) were estimated by GPC in THF by using a calibration curve of poly (styrene) standard. c) The molar ratio of **M1** to **M2** was estimated from the ^{1}H NMR. d) Lifetime of SPNs monitored at 515 nm. e) Lifetime of SPNs monitored at 704 nm. f,g) Absolute quantum efficiency at 515 nm and 704 nm, respectively.

Table S2. Parameters of stern-volmer equation under different oxygen contents.

	Fixed cell				Living cell	
**Oxygen level**	R_i	R_i^0/R_i	τ_i/ns	τ_0/τ_i	R_i	τ_i/ns
**N_2**	1.12	1	122	1.00	1.15	121
**2.5% O_2**	1.04	1.08	115	1.06	1.04	117
**5% O_2**	0.75	1.49	105	1.15	0.87	108
**10% O_2**	0.60	1.87	96	1.26	0.70	98
**15% O_2**	0.51	2.20	90	1.34	0.58	93
**21% O_2**	0.44	2.55	73	1.68	0.51	80

Table S3. The intracellular oxygen contents [O_2] in living cells treated with **P1** under different extracellular oxygen contents.

Extracellular [O_2]/%	5	10	15	21
Intracellular [O_2]/% calculated based on the ratiometric luminescence imaging in living cells	4	8	13	16
Intracellular [O_2]/% calculated based on phosphorescence lifetime imaging in living cells	4	8	11	18

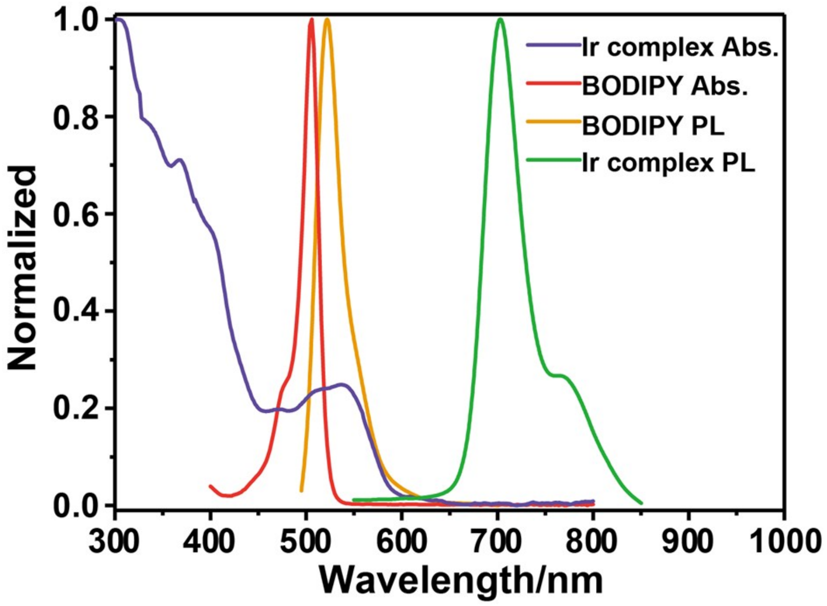

Fig S1. The absorption and PL spectra of monomers.

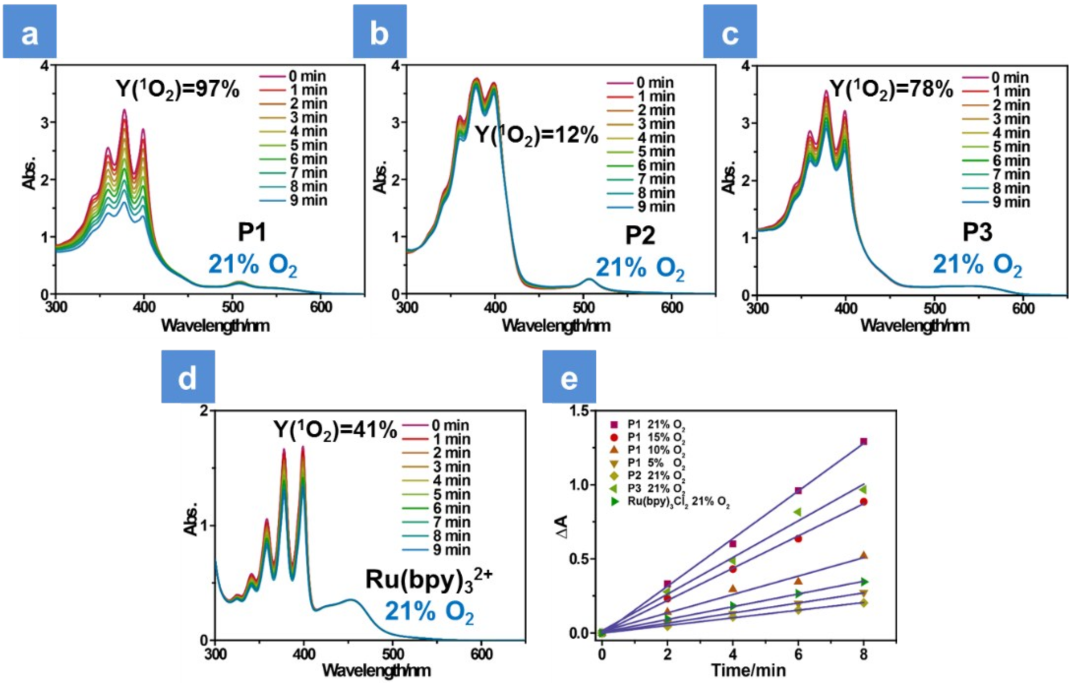

Fig S2. UV-Vis absorption of the ABDA solution mixed with **P1**, **P2**, **P3** and $Ru(bpy)_3^{2+}$ after irradiation by xenon lamp. The irradiation time interval was 1 min. a-c) Solution of **P1**, **P2** and **P3** under oxygen contents of 21%, respectively. d) Solution of $Ru(bpy)_3^{2+}$ under oxygen content of 21%. e) Plots of the absorption difference at 400 nm as a function of light irradiation time in different conditions.

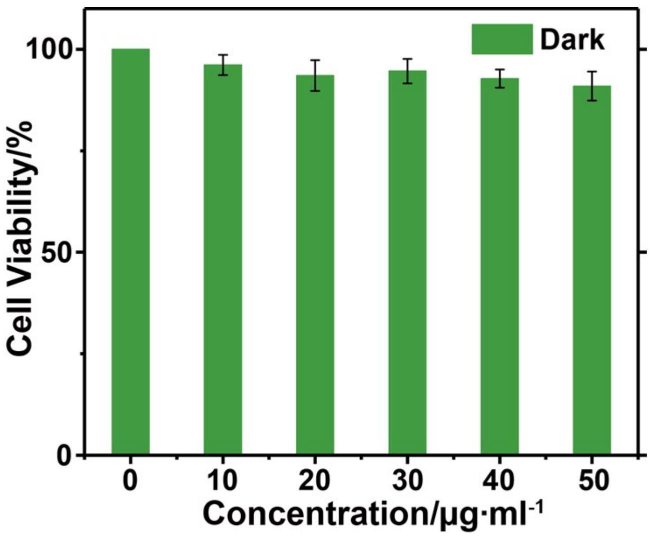

Fig S3. MTT assay of Hela cells incubated with **P1** in darkness.

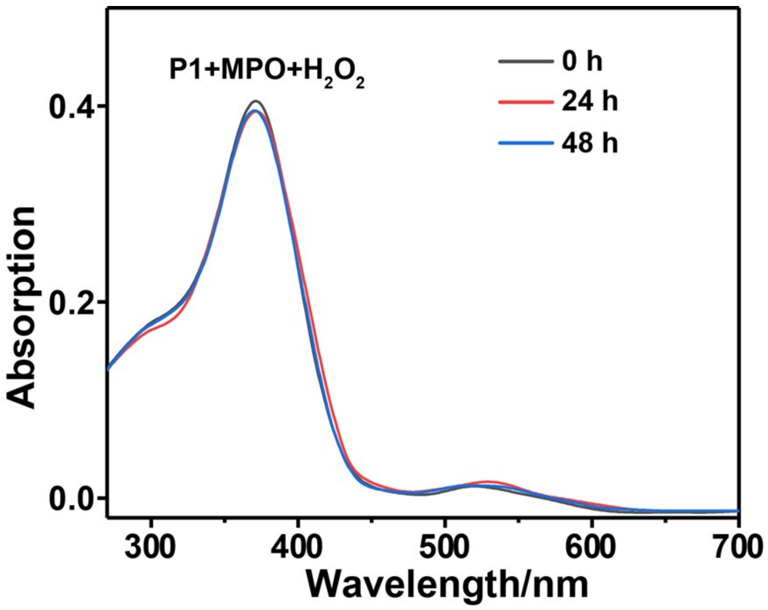

Fig S4. Absorption spectra of **P1** (20 μg/mL) in PBS containing NaCl (150 mM) together with the mixture of MPO (100 μg/mL) and H_2O_2 (600 μM) at 37 °C for 0, 24, and 48 h.

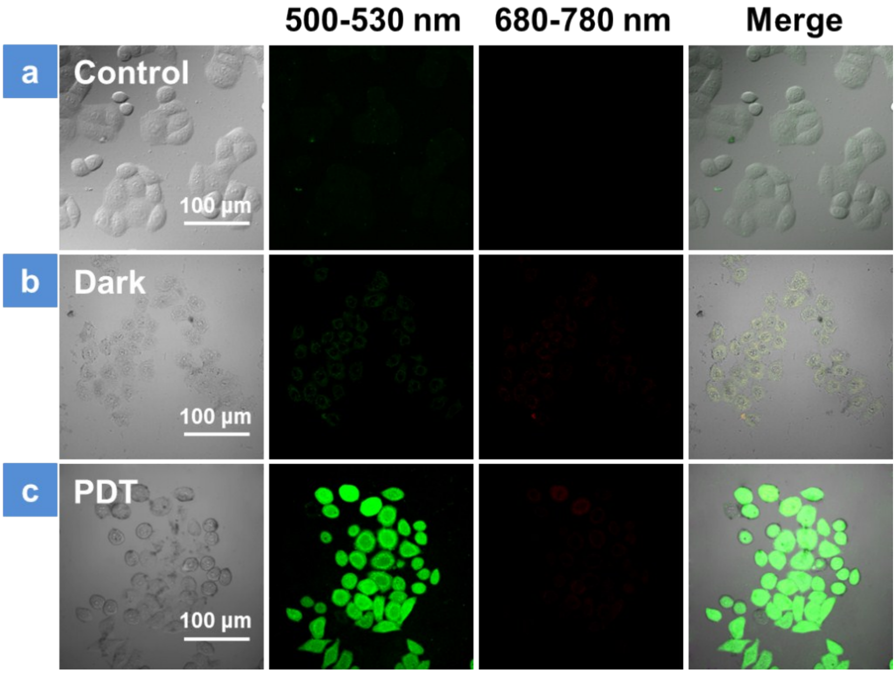

Fig S5. ROS observation via laser scanning confocal microscopy. a) Control group. b) Hela cells incubated with **P1** and stained with DCFH-DA were exposed in darkness. c) Hela cells incubated with **P1** and stained with DCFH-DA were exposed under light irradiation.

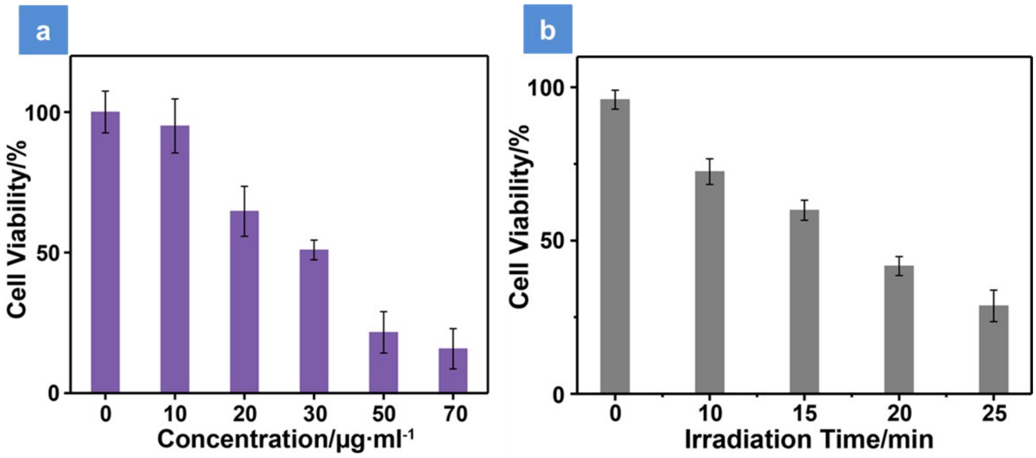

Fig S6. Cytotoxicity assessment of **P1**. a) MTT assay of Hela cells incubated with different concentrations of **P1** (0, 10, 20, 30 50 and 70 μg/mL) under 10 min irradiation. b) MTT assay of Hela cells incubated with **P1** (50 μg/mL) under different irradiation times (0, 2.5, 5, 10 and 15 min).

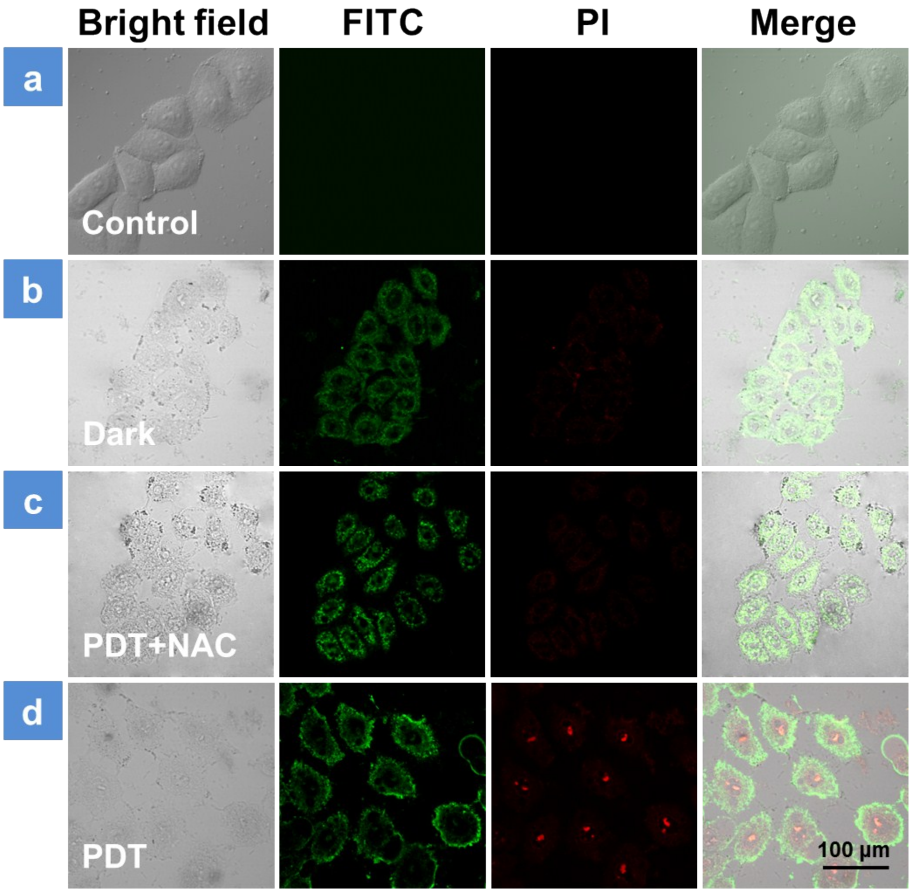

Fig S7. Confocal fluorescence images of Annexin V-FITC/PI stained Hela cells treated in different conditions. a) Hela cells were cultured with PBS only. b) Cells were incubated with **P1** without irradiation. c) Hela cells were cultured with NAC and **P1**, and then exposed under 475 nm xenon lamp. d) Hela cells incubated with **P1** were exposed under 475 nm xenon lamp.

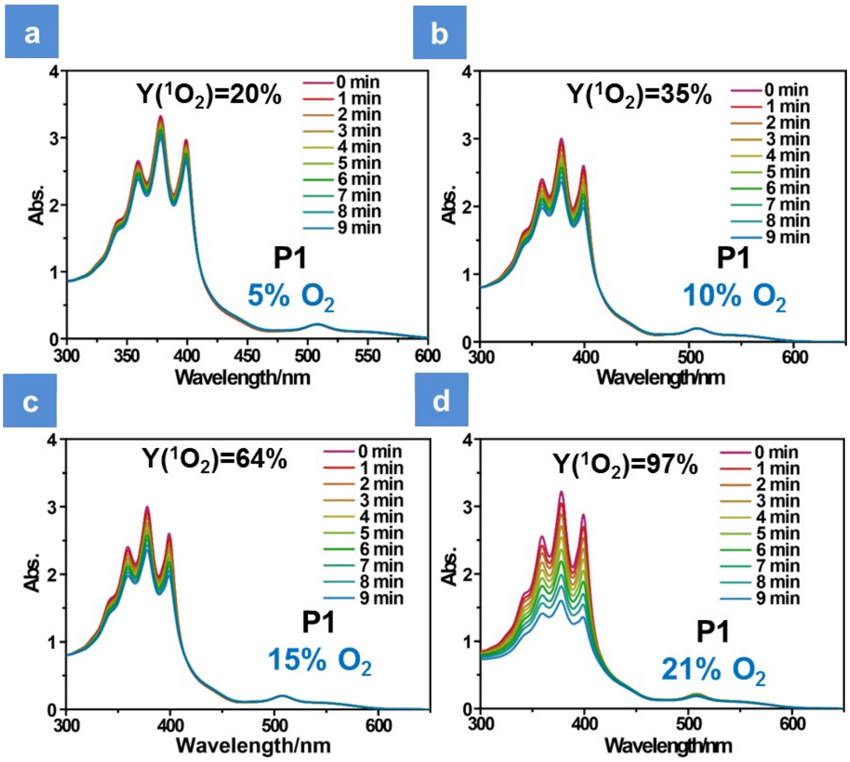

Fig S8. UV-Vis absorption of ABDA solution mixed with **P1** after irradiation by xenon lamp. The irradiation time interval was 1 min. a-d) Solution of **P1** and ABDA exposed under xenon lamp in oxygen contents of 5, 10, 15 and 21% respectively.

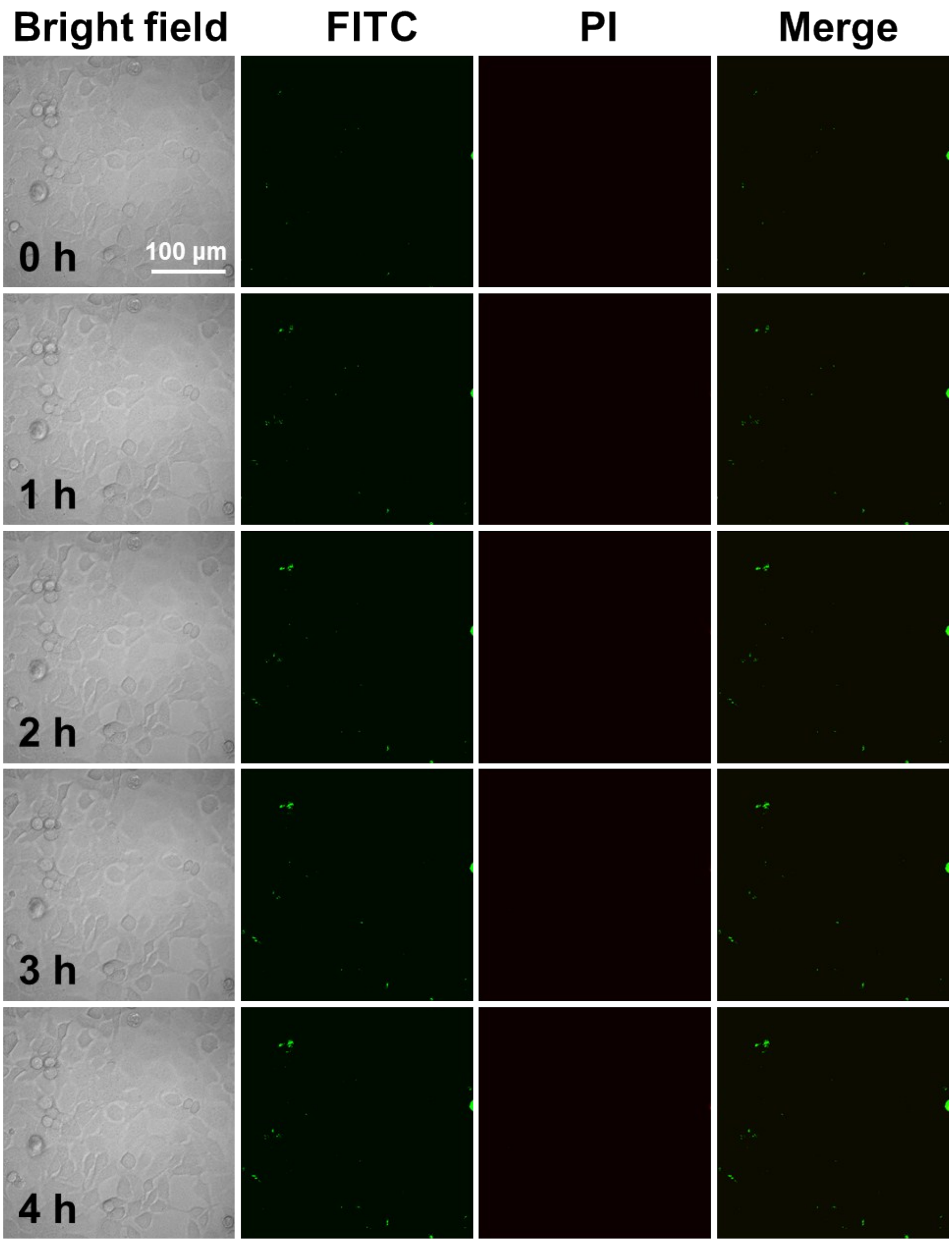

Fig S9. Time series model of CLSM images for Hela cells. The cells were cultured at 37 °C for 4 h and then stained with Annexin V-FITC/PI under normoxia condition. The signal in green channel for Annexin V-FITC (λ_{ex} = 488 nm, λ_{em} = 500–560 nm) and that in red channel for PI (λ_{ex} = 488 nm, λ_{em} = 600–680 nm) were collected. The images were acquired in 4 h with an interval of 1 h after irradiation.

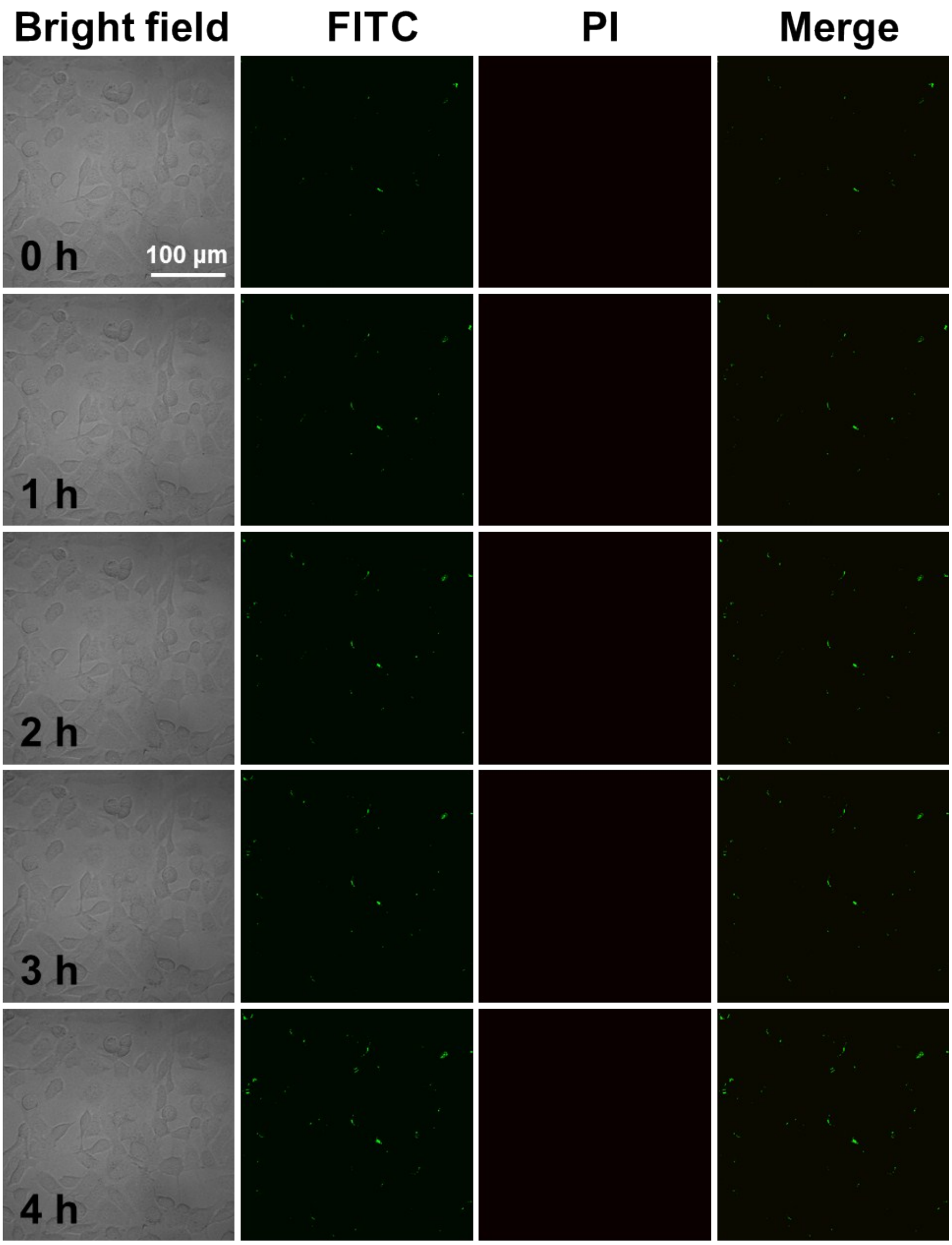

Fig S10. Time series model of CLSM images for Hela cells. The cells were cultured with **P1** at 37 °C for 4 h and then stained with Annexin V-FITC/PI under normoxia condition. The signal in green channel for Annexin V-FITC (λ_{ex} = 488 nm, λ_{em} = 500–560 nm) and that in red channel for PI (λ_{ex} = 488 nm, λ_{em} = 600–680 nm) were collected. The images were acquired in 4 h with an interval of 1 h after irradiation.

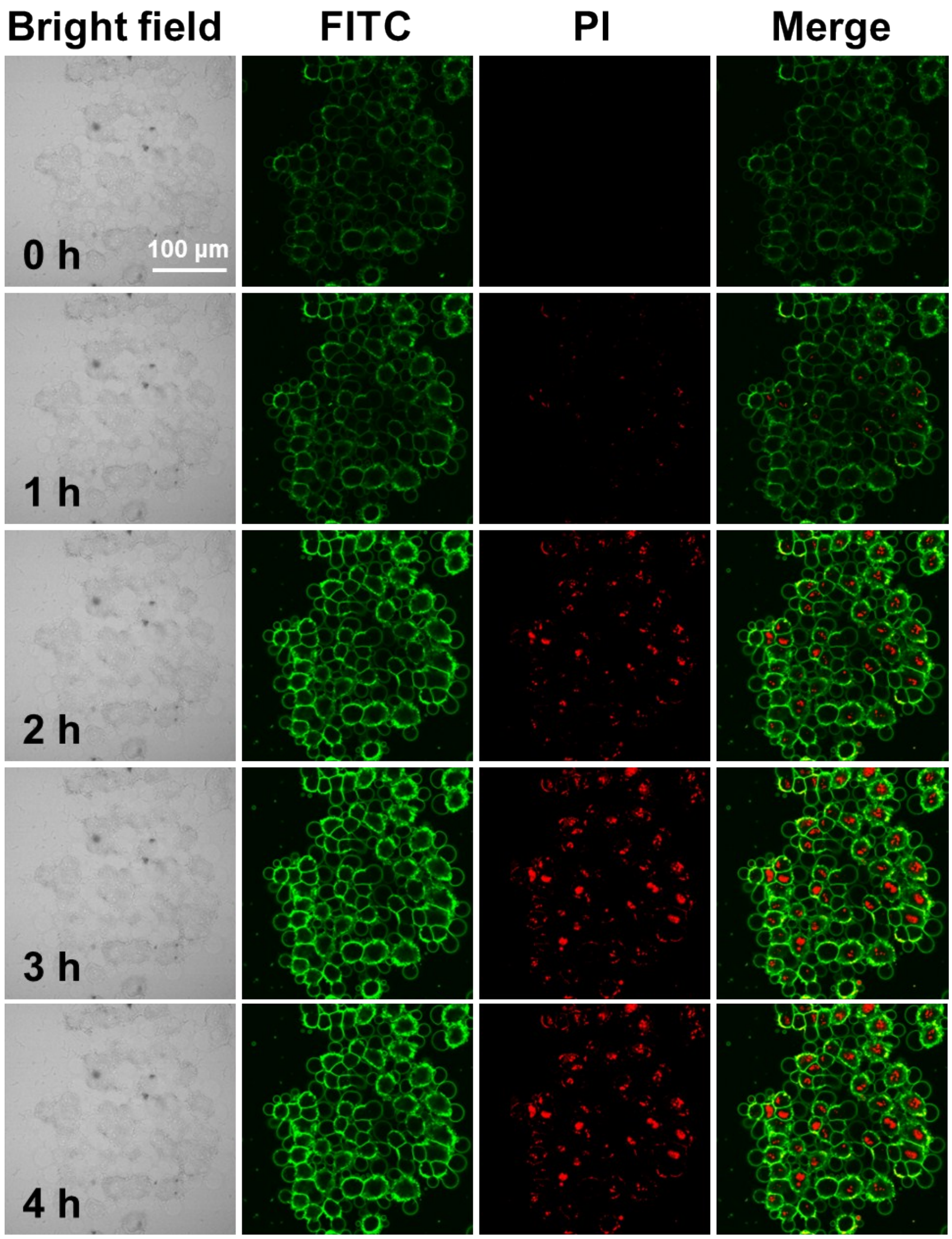

Fig S11. Time series model of CLSM images for Hela cells. The cells were cultured with **P1** at 37 °C for 4 h under normoxial condition and were exposed under 475 nm irradiation for 15 min before Annexin V-FITC/PI agent was added. The signal in green channel for Annexin V-FITC (λ_{ex} = 488 nm, λ_{em} = 500–560 nm) and that in red channel for PI (λ_{ex} = 488 nm, λ_{em} = 600–680 nm) were collected. The images were acquired in 4 h with an interval of 1 h after irradiation.

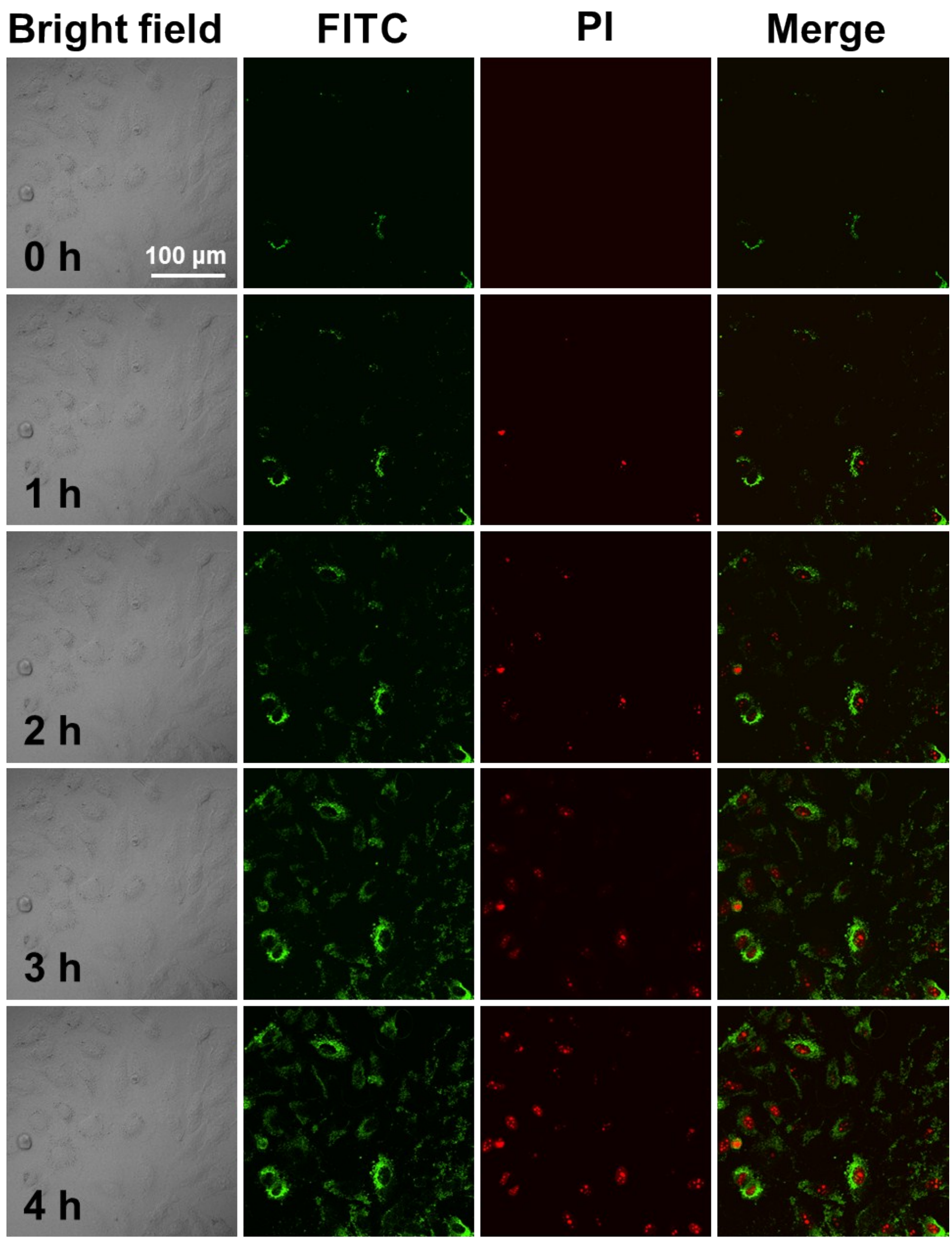

Fig S12. Time series model of CLSM images for Hela cells. The cells were cultured with **P1** at 37 °C for 4 h under hypoxia condition and were exposed under 475 nm irradiation for 15 min before Annexin V-FITC/PI agent was added. The signal in green channel for Annexin V-FITC (λ_{ex} = 488 nm, λ_{em} = 500–560 nm) and that in red channel for PI (λ_{ex} = 488 nm, λ_{em} = 600–680 nm) were collected. The images were acquired in 4 h with an interval of 1 h after irradiation.

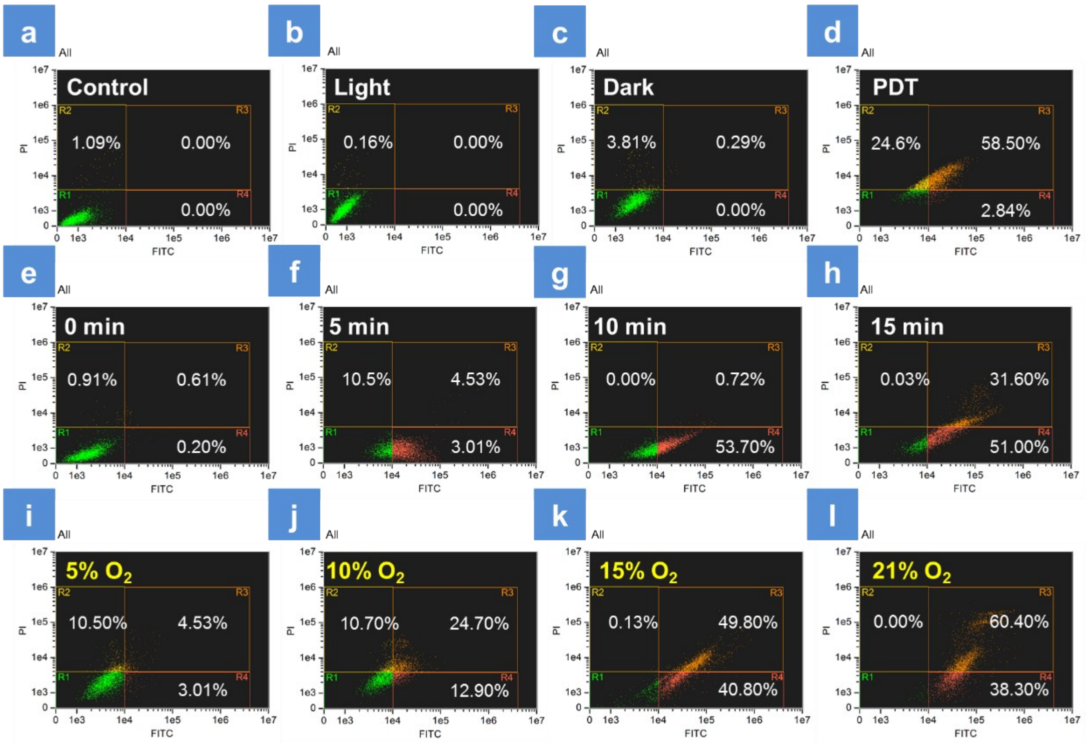

Fig S13. Flow cytometer analysis of Hela cells treated in different conditions a-d) Cells were cultured with PBS only, light irradiation only, **P1** without irradiation and **P1** under irradiation, respectively. e-h) Cells incubated with **P1** were treated with 475 nm xenon lamp in normoxia for 0, 5, 10 and 15 min respectively. i-l) Cells incubated with **P1** were exposed under 475 nm xenon lamp for 15 min with oxygen contents of 5, 10, 15 and 21%, respectively.

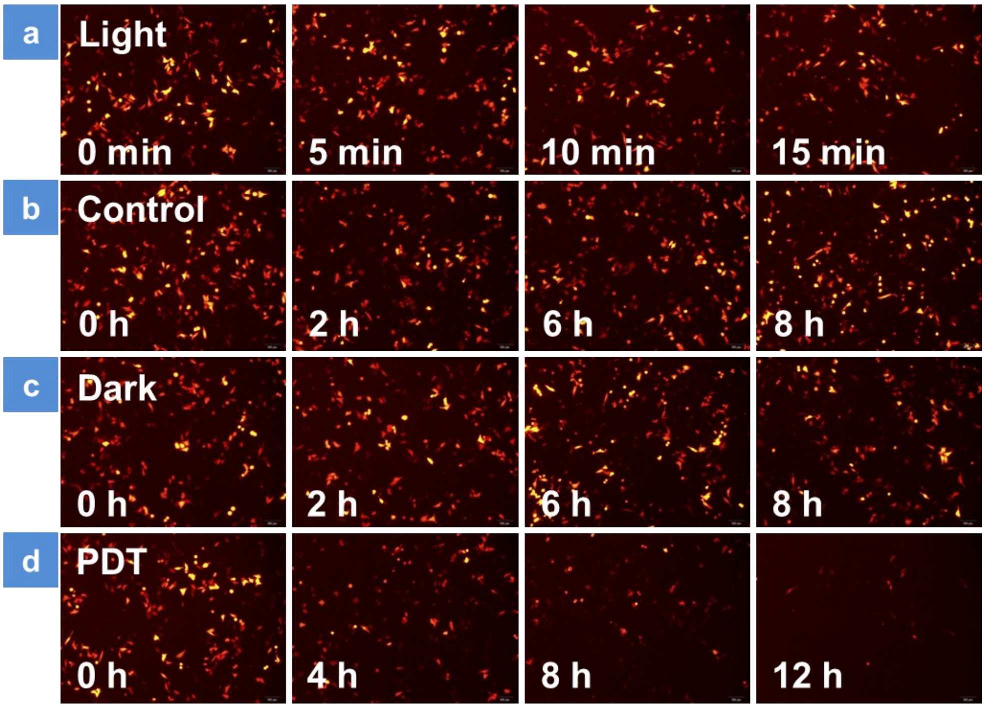

Fig S14. Inverted fluorescence microscope images with DsRed transfected Hela cells. a) Cells were cultured with PBS only. b) Cells were treated with light irradiation only. c) Cells were incubated with **P1** for 4 h without irradiation. d) Cells were incubated with **P1** for 4 h and then exposed under 475 nm xenon lamp.

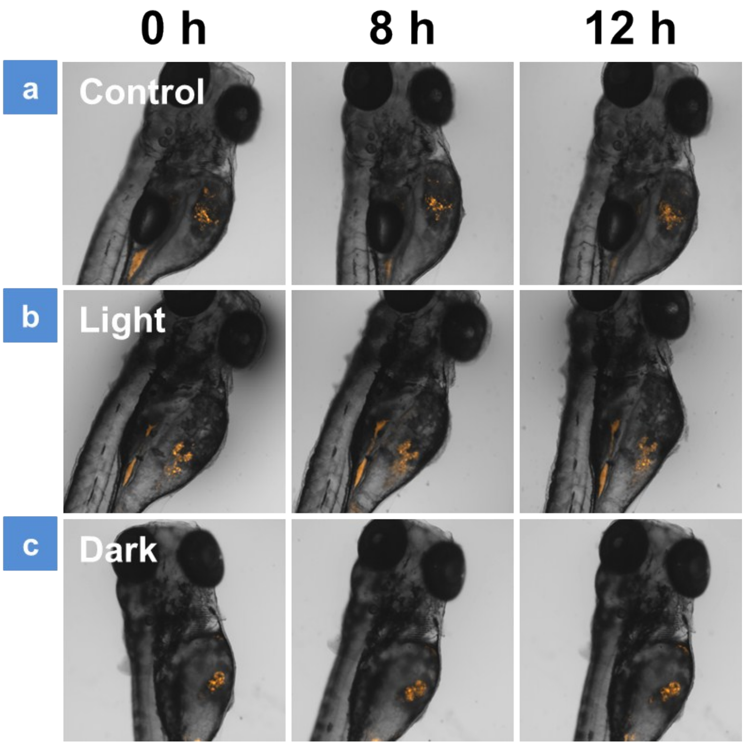

Fig S15. Representative CLSM images of xenograft zebrafish under different conditions. a-c) The zebrafish was treated with PBS only, light irradiation only and **P1** injection without irradiation, respectively. The fluorescence signal was collected between 570 and 590 nm.

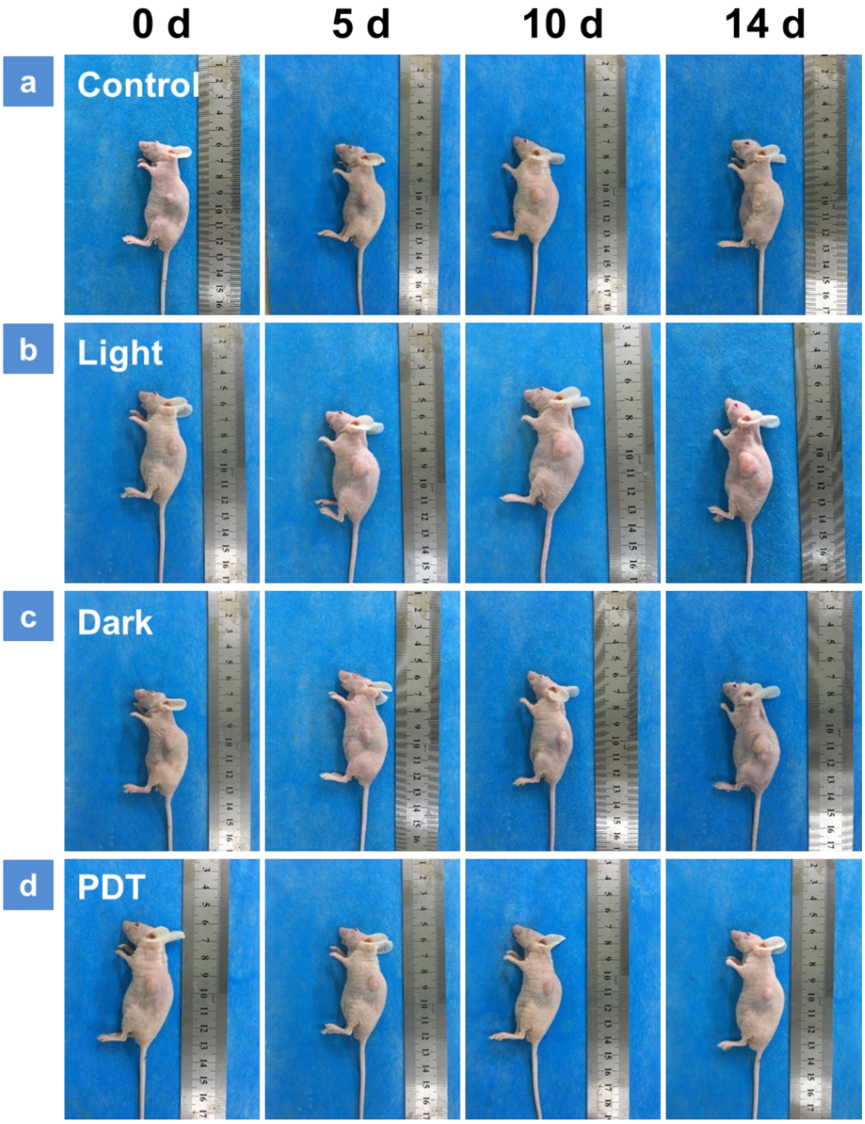

Fig S16. Photos of mice treated in different situations. a-d) The tumor-bearing mice were treated with PBS only, light irradiation only, **P1** without irradiation and **P1** under irradiation, respectively.

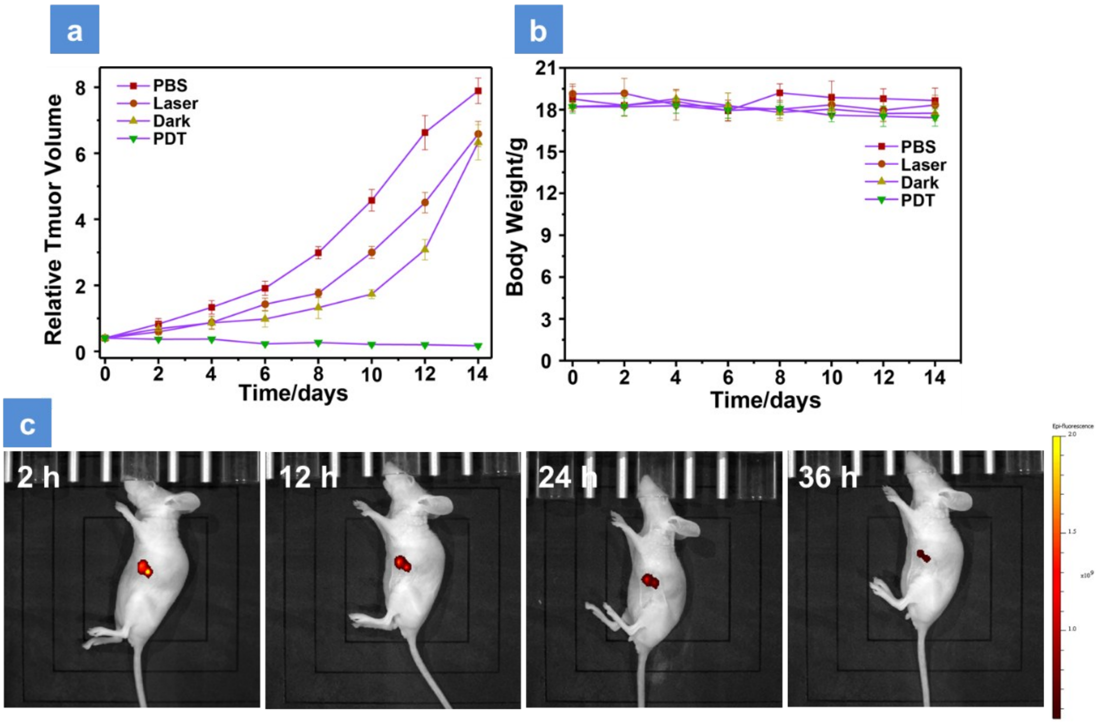

Fig S17. a) Relative tumor volume of mice treated in different situations within two weeks. b) Time-dependent body weight curves of different experimental groups after different treatments. c) *In vivo* fluorescence imaging of tumor-bearing mice at 2, 12, 24 and 36 h post injection of **P1**.
